# Ultra-rapid cryo-EM data acquisition method enabled by continuous recording based beam image shift

**DOI:** 10.1038/s41467-026-74373-6

**Published:** 2026-06-12

**Authors:** Qi Yang, Xiaojun Huang, Chunling Wu, Yan Zeng, Xinzheng Zhang

**Affiliations:** 1 State Key Laboratory of Biomacromolecules, Institute of Biophysics, Chinese Academy of Sciences, Beijing, China; 2 University of Chinese Academy of Sciences, Beijing, China; 3 Center for Biological Imaging, Core Facilities for Protein Science, Institute of Biophysics, Chinese Academy of Sciences, Beijing, China; 4 National Multi-mode Trans-scale Biomedical Imaging Center, Beijing, China; 5 Interdisciplinary Center for Biointelligence, Institute of Biophysics, Chinese Academy of Sciences, Beijing, China

**Keywords:** Cryoelectron microscopy, Cryoelectron tomography

## Abstract

Cryo-electron microscopy (cryo-EM) data acquisition is time-intensive given that a large amount of data is required to obtain a high-resolution reconstruction. Here, we eliminate camera-induced delay time by continuously recording during beam-image shift acquisition using a method called Continuous Recording Beam-Image Shift (CR-BIS). The utilization of CR-BIS with K3 and Falcon 4 direct electron detectors and conventional data acquisition conditions enables the acquisition of ~34,000 micrographs and ~1,000 tilt series per 24 h in single-particle analysis mode and cryo-electron tomography mode, respectively. Three-dimensional reconstructions of single-particle and tomographic datasets show that CR-BIS accelerates data collection and maintains data quality. CR-BIS is broadly applicable for efficient high-resolution cryo-EM since it can be implemented into existing acquisition software through scripting and it does not require hardware modification.

## Abstract (web summary)

The authors report continuous recording beam-image shift (CR-BIS), a continuous recording strategy that overcomes camera delays to markedly accelerate cryo-EM data acquisition, improving throughput for single-particle analysis and cryo-electron tomography without loss of quality.

## Introduction

Cryo electron microscopy (cryo-EM) has become a key technique in structural biology. It is broadly applied to high-resolution structure determination, in situ visualization of cellular ultrastructures, and drug discovery. However, high-resolution structural determination using cryo-EM depends on the acquisition of large datasets^1^. Despite advances in hardware and improvements in the efficiency of cryo-EM through high-throughput data acquisition methods, the high cost and limited instrument time continue to pose significant challenges. Therefore, enhancing data collection efficiency remains a crucial objective for the field.

Traditional cryo-EM data acquisition utilizes the stage translation approach through mechanical stage movements and stabilization at each target, which results in substantial idle time. This reduces the efficiency of the data acquisition to less than 3000 images per day^2^. Currently, the application of beam-image shift (BIS) replaces most stage movements and significantly improves the efficiency of data throughput. Although BIS was proposed over a decade ago, early applications were hindered by BIS-induced coma and astigmatism under sub-optimal conditions, which decreased the final image quality^3,4^. Wu and colleagues measured and calibrated the beam tilt and astigmatism induced by beam shift and demonstrated that BIS could be applied reliably in single-particle analysis (SPA) within a radius of ~10 μm^5^. Similar strategies have been used in SerialEM^6,7^, JADAS^8^, and EPU^9,10^ to actively correct the aberration when BIS mode is performed with a mature and convenient pipeline. When coupled with new-generation high-end EM cameras (e.g., Gatan K3 or Thermo Scientific Falcon 4)^11,12^, this approach typically facilitates the data collection of ~10,000 micrographs within 24 h^13,14^.

BIS mode also improves the efficiency of tilt series data collection for cryo-electron tomography (cryo-ET). In cryo-ET, a series of images is collected from the same region at different tilt angles, which are combined to reconstruct a three-dimensional tomogram. Because each tilt requires stage movement, stabilization, and tracking, the acquisition process is inherently time-consuming. Several groups implemented BIS for cryo-ET in practice^14–16^. For each tilt angle, images that belong to different tilt series are acquired successively using BIS mode. The parameters of BIS were carefully adjusted according to the geometry of the sample, and included the shift distance and defocus. Beam image-shift electron cryo-tomography yields near-atomic resolution for purified protein^15^. Parallel cryo-electron tomography (PACE-Tomo) uses a geometrical sample model to predict the effect of tilting across many target positions, which emphasizes acquisition throughput and robustness on real lamellae^16^. PACE-Tomo presents a precision alignment for all tilt angles within ±50 nm and improves the data acquisition efficiency to ~300 tilt series per day^16^, which represents a major step toward efficient tomography.

While the application of BIS has markedly improved data collection efficiency for SPA and cryo-ET, the pursuit of high-resolution structures in structural biology requires the acquisition of an exceptionally large dataset owing to the nonlinear relationship between resolution and demanding data size^1^. This demand poses a particularly formidable challenge in in situ cryo-ET, where the throughput remains substantially lower than that of SPA^17^. In addition, the number of particles of a specific protein complex in an in situ dataset is determined by its natural abundance. To date, the few in situ structures determined at near-atomic resolution predominantly belong to highly abundant targets, such as the ribosome^18–20^. The high cost of a premium microscope makes large-scale tomographic campaigns exceptionally resource-intensive. Despite the gains afforded by BIS, the data collection rate continues to be a critical bottleneck, which limits the resolution and general applicability of cryo-ET for many biological targets.

Here, we present a Continuous Recording Beam-Image Shift (CR-BIS) framework that eliminates the inherent per-exposure delays in conventional acquisition methods. Under standard acquisition conditions, CR-BIS enables the collection of 34,000–44,000 micrographs per 24 h for SPA (depending on the magnification) and ~1000 tilt series per 24 h for cryo-ET. Compared with conventional data collection methods, this represents a twofold to threefold and threefold increase in throughput for SPA and cryo-ET, respectively^5,15,16^. Successful implementation of CR-BIS in SerialEM^6,7^ does not require hardware modification. Furthermore, it can be integrated into other data acquisition pipelines through scripting, which provides a general solution for high-throughput SPA and cryo-ET studies. Faster collection reduces costs, increases accessibility to high-resolution SPA, and accelerates in situ tomography. More efficient acquisition facilitates large-scale studies in structural genomics and strengthens the application of cryo-EM in pharmaceutical research.

## Results

### The effective acquisition ratio reduced by the camera delay time

**Fig. 1:**
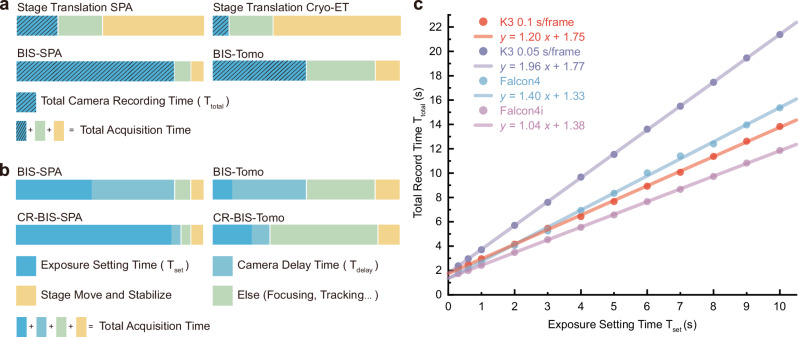
Time allocation and acquisition efficiency in cryo-EM data collection. **a** Schematic comparison of time allocation for conventional stage translation, beam-image shift (BIS), and cryo-electron tomography (cryo-ET) acquisition. The dashed blue region indicates the total camera recording time (T_total_), which includes both the exposure period and camera-induced delay. The proportion of stage movement time is shown in yellow, while other time components, such as focusing and tracking, are shown in green. The ratio of T_total_ to the total acquisition time is referred to here as the conventional acquisition efficiency. **b** Decomposition of T_total_ into the user-defined exposure setting time (T_set_, dark blue) and the camera delay time (T_delay_, light blue). The proportion of stage movement time is shown in yellow, while other time components, such as focusing and tracking, are shown in green. The effective acquisition ratio (EAR) is defined as the ratio between T_set_ and the total acquisition time, representing the true fraction of time spent on electron exposure. **c** Relationship between T_set_ and T_total_ across different cameras and frame rates. Each data point is the average of three replicate measurements (*n* = 3). Red and purple lines show T_total_ as a function of T_set_ for the K3 direct electron detector at frame times of 0.1 s/frame and 0.05 s/frame, respectively. Blue and pink lines show the corresponding relationships for the Falcon 4 and Falcon 4i direct electron detectors, respectively.

The key steps of automated cryo-EM data acquisition include stage movement, low- and high-magnification alignments prior to imaging, defocus adjustment, and exposure of target areas. The central principle of the BIS strategy is to compress the duration of non-exposure steps and maximize the fraction of actual exposure within the overall workflow, which is essential for high-throughput data collection. A conventional measure of acquisition efficiency is the ratio between the total camera recording time (T_total_) and total acquisition time (the entire data acquisition process). The time allocation for each major step was plotted, with the conventional acquisition efficiency for different methods shown in Fig. 1a. The traditional stage-translation approach relies on mechanical stage movements and stabilization at each target position, which results in substantial idle time and a conventional acquisition efficiency of less than 20%. The introduction of BIS methods for SPA^4,5^ has replaced mechanical movements with near-instantaneous beam deflection, which reduces field-of-view switching to milliseconds and increases the conventional acquisition efficiency to ~80%. Similarly, the application of BIS in cryo-ET improves the conventional acquisition efficiency from less than 10 to ~50%. This conventional acquisition efficiency was calculated using T_total_ (dashed area in Fig. 1a), which includes exposure time and camera-induced delay and does not reflect the true effective exposure time.

In standard acquisition, T_total_ for a single exposure consists of the user-defined exposure setting time (T_set_) and an additional camera delay time (T_delay_) (shown as the dark blue and light blue areas in Fig. 1b, respectively). For more accurate quantification of data acquisition efficiency, we defined the effective acquisition ratio (EAR) as the ratio between the exposure setting time and the total acquisition time. Because the conventional metric does not account for camera-induced delays, it overestimates the fraction of time spent on actual electron exposure. When camera delay was explicitly considered (Fig. 1b), the actual EAR for BIS-based acquisition was reduced to ~40% for SPA and ~10% for cryo-ET, which indicates substantial inefficiencies. Using the continuous recording (CR) strategy introduced below, we eliminated per-exposure camera delays and increased the EAR to ~90% for SPA and ~20% for cryo-ET, as shown in Fig. 1b.

We quantified the relationship between T_set_ and T_total_. The variation of T_total_ with the change in T_set_ of different cameras and frame times is shown in Fig. 1c. For the K3 direct electron detector, the delay time includes an intercept term and a slope term, which is related to the frame rate (red and purple lines in Fig. 1c). The K3 direct electron detector operates internally at 1500 Hz, with a maximum readout of 75 frames per second (fps)^11,21^. The slope of the K3 direct electron detector increased from 1.20 to 1.96 when the frame rate changed from 10 to 20 fps (corresponding to frame time 0.1 s/frame to 0.05 s/frame). A higher frame rate benefits motion correction, although it results in a larger slope term (Fig. 1c). Despite the variation in the slope term for K3, the intercept term remains nearly constant (~ 1.7 s). In electron-event representation (EER) mode, the internal frame rates and frame rates to storage of Falcon 4 and Falcon 4i are ~250 and 320 fps, respectively^12,22,23^. A fixed and unchangeable frame rate resulted in constant slopes for Falcon 4 (~ 1.40) and Falcon 4i (~1.04). The intercept terms for Falcon 4 (~ 1.33 s) and Falcon 4i (~1.38 s) remained almost unchanged during multiple tests. Meanwhile, the K2 direct electron detector produced a similar relationship between T_total_ and T_set_, although the slope term was larger (Supplementary Fig. 1). These results indicate that T_delay_ is the sum of the slope term and intercept term. Therefore, it is critical to address the camera delay time to enhance the efficiency of cryo-EM data acquisition and improve the EAR.

### The principle and workflow of CR-BIS

**Fig. 2:**
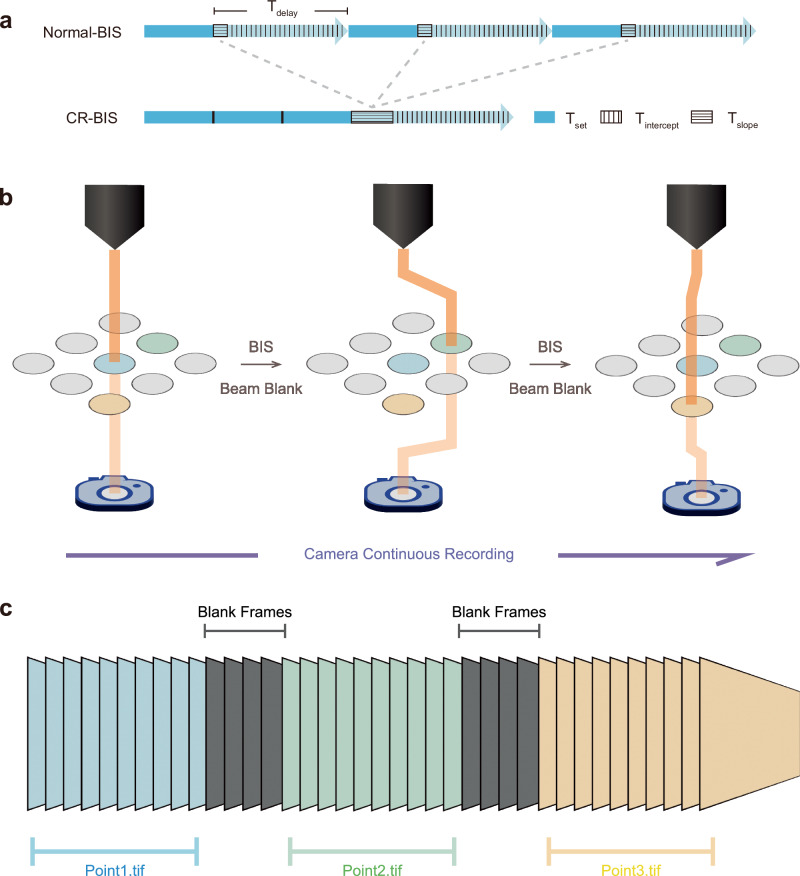
Principle and workflow of CR-BIS. **a** Schematic illustration of the acceleration principle underlying CR-BIS. Each exposure (exposure setting time, T_set_, dark blue region) is followed by a camera delay time (T_delay_, light blue region). T_delay_ consists of an exposure-independent intercept term (T_intercept_, vertical stripes region) and an exposure-dependent slope term (T_slope_, horizontal stripes region). The number of repeated T_intercept_ events can be substantially reduced through the replacement of multiple short exposures with a single long exposure. **b** Flowchart of the CR-BIS data acquisition procedure. Taking three target points (blue, green, yellow) on a grid as an example, multiple short exposures within a single beam-image shift (BIS) group are replaced by a single long continuous exposure. The electron beam (orange line) is blanked immediately after each exposure, then repositioned via BIS to the next target, and finally restored to initiate the next acquisition cycle. During the acquisition of a BIS target group, the camera remains in continuous recording mode. **c** Composite image stack generated by CR-BIS, containing all target images and beam-blanked frames acquired during continuous recording. Frames containing images of different target points are shown in blue, green, and yellow, respectively, while frames corresponding to beam blanking are shown in black. This stack is subsequently split using an intensity- and blank-frame-position-based script, which identifies blank frames and exports individual image stacks corresponding to each target location.

The basic principle of resolving camera-related delay times is illustrated in Fig. 2a. In this schematic, T_delay_ is divided into two components: an exposure-independent intercept term (T_intercept_, vertical striped region) and an exposure-dependent slope term (T_slope_, horizontal striped region). It should be noted that Fig. 2a does not represent the exact temporal order of events during an exposure. In practice, intercept-related delays may occur before and after the nominal exposure period.

The number of repeated T_intercept_ events can be substantially reduced through the replacement of multiple short exposures with a single long exposure. For example, three short exposures incur three separate intercept delays, whereas a single long exposure incurs only one (Fig. 2a), which significantly increases the EAR. By contrast, the T_slope_ increases with longer exposure setting time. In intermittent acquisition, these slope-derived delays occur discretely after each exposure; in long CR, they are effectively consolidated into a single delay period at the end of the recording.

To mitigate the impact of T_slope_, we implemented a script that triggers stage movement ahead at the end of long recordings when the camera and control software remain unresponsive owing to T_delay_. This strategy effectively utilizes the overall delay period, with particular benefit for the exposure-time-dependent T_slope_ component.

The CR-BIS workflow is depicted in Fig. 2b. For each focus group, focusing is performed first, followed by continuous recording of all target positions. Following exposure at each position, the beam was blanked during the beam shift to the next target, resulting in blanked frames, which appeared with low intensity in the recorded stack. For CR-BIS-Tomo, all targets at a certain tilt angle were collected using CR-BIS. To estimate the ΔZ error arising from surface unevenness of the lamella, the first five tilts were acquired without CR mode (Supplementary Fig. 2). For the remaining tilts, all target positions at each tilt angle were collected using CR-BIS with the updated ΔZ correction. Under commonly used cryo-ET acquisition conditions (tilt step of 2° and 3°, tilt range of ±38° and ±60°, and dose of 3 and 4 e^−^/Å^2^ per tilt), the alignment results from these initial five tilts provide a reliable estimation of ΔZ, as we tested. The number of initial non-CR tilts is user-configurable in the provided scripts and can be increased when required by specific experimental conditions.

The total exposure setting time (T_total set_) of multiple targets for CR-BIS is given by Eq. (1):

1 $${{{\rm{T}}}}_{{{\rm{total}}}\; {{\rm{set}}}}=\left({{{\rm{T}}}}_{{{\rm{set}}}\; {{\rm{per}}}\; {{\rm{point}}}}+{{{\rm{T}}}}_{{{\rm{blank}}}}\right)\times {{\rm{point}}}\; {{\rm{number}}}+{{{\rm{T}}}}_{{{\rm{buffer}}}}$$

The calibrated formula is unaffected by fluctuations in the slope or intercept terms. The formulas during our tests were ${{{\rm{T}}}}_{{{\rm{total}}}\; {{\rm{set}}}}=\,\left({{{\rm{T}}}}_{{{\rm{set}}}\; {{\rm{per}}}\; {{\rm{point}}}}+\,0.22\,{{\rm{s}}}\right)\,\times {{\rm{point}}}\; {{\rm{number}}}\,+\,0.6\,{{\rm{s}}}$ for the K3 direct electron detector and ${{{\rm{T}}}}_{{{\rm{total\; set}}}}=\left({{{\rm{T}}}}_{{{\rm{set}}}\; {{\rm{per}}}\; {{\rm{point}}}}+0.26\,{{\rm{s}}}\right)\times {{\rm{point}}}\; {{\rm{number}}}+1.2\,{{\rm{s}}}$ for the Falcon 4 direct electron detector. The T_blank_ time including beam blank/unblank and BIS varies depending on the type of cryo-EM models. Here, we used the continuous mode in serialEM. Transition from the normal mode to the continuous mode involves differing durations in variable cryo-EM models. Thus, we recommend that users add a buffer time (T_buffer_)to ensure that the total duration captures each target point. Pre-calibration is required before using CR-BIS for specific cryo-EM models (especially for the blank time). Detailed pre-calibration procedures are described in “Methods”.

As shown in Fig. 2c, the CR-BIS method records all frames corresponding to the target points within a single, continuous data stack. Although captured in one acquisition, the frames of the individual targets were separated by the blank frames generated during the beam blanking periods. According to the fluctuation of the intensity in each frame of the unabridged stack, a threshold was set to identify the blank frames from the output files for removal. Meanwhile, the stack was easily split into individual groups based on the blank-frame positions, which were considered boundaries. An example intensity record of CR-BIS output was shown in Supplementary Fig. 3. During each beam blank or unblank, at most one moderate frame was observed, where the intensity was larger than the blank frames and smaller than the average intensity of target unblank frames. The moderate frames were caused by beam blanking/unblanking processes and may be added randomly into the split groups based on the threshold, which results in a maximum fluctuation of one frame in the number of frames for the output segmented images. Our test datasets demonstrate that fluctuations in the frame count do not compromise data quality. Furthermore, frames with moderate intensity can be fully removed through the use of another script.

### Ultra-rapid speed performance of CR-BIS

To evaluate the performance of the speed improvement of CR-BIS, we collected SPA and tomographic datasets and compared acquisition speeds using our BIS^5^ and CR-BIS. As shown in Table 1, the total acquisition time of our conventional BIS-SPA was twice as long as CR-BIS-SPA (where SPA used 2 s exposure per position, 0.1 s/frame, and 100 targets per focus group). For cryo-ET, CR-BIS-Tomo reduced acquisition time by ~twofold compared to BIS-Tomo using K3 and Falcon 4 direct electron detectors, and by ~2.7-fold compared to the PACE-Tomo protocol^16^ with similar parameters (~ 4.5 min per tilt series using a K3 direct electron detector, tilt range of 120°, tilt step of 3°, 0.5 s per target, and 0.1 s/frame). The average acquisition rate of our control BIS was slightly higher than that reported for PACE-Tomo. The only difference between our control BIS and CR-BIS setups was the CR mode. The improvement ratio for K3 and Falcon 4 direct electron detectors increased with the decrease of exposure setting times per point (Supplementary Table 1). For example, CR-BIS-SPA achieved a 3.8-fold improvement in acquisition speed compared to our conventional BIS-SPA using the K3 direct electron detector, with 0.5 s exposure per position and 0.1 s/frame (more detailed test parameters shown in Supplementary Table 1). This indicates that the acceleration of CR-BIS is more significant when the exposure setting time for each target point is shorter than 2 s, which is particularly helpful for cryo-ET data collection with a very low exposure dose (usually around 3 e^−^/Å^2^) for each tilt angle. The improvement in the EAR after applying CR-BIS was ~90% for SPA and ~20% for cryo-ET (Fig. 1b).

**Table 1:** Comparison of our BIS and CR-BIS total acquisition times in SPA and cryo-ET

	Detector	Acquisition parameters	BIS	CR-BIS	Improvement ratio
SPA	K3	100 points/group, 0.1 s/frame, 2 s/point	547 s	254 s	2.15
	Falcon 4	25 points/group*4, 2 s/point	566 s	368 s	1.53
Cryo-ET	K3	25 points/group, 0.1 s/frame, 0.5 s/point	3.25 min/ts	1.64 min/ts	1.98
	Falcon 4	25 points/group, 0.5 s/point	2.76 min/ts	1.34 min/ts	2.05

Total acquisition times were evaluated on the K3 and Falcon 4 direct electron detectors using conventional BIS and CR-BIS. Parameters include the number of beam-shift positions, exposure setting time per point, and frame rate. CR-BIS consistently accelerated acquisition across modalities, achieving 1.5–2.1× speed improvements.

High-resolution structural analysis at near-atomic detail relies on a sufficiently high signal-to-noise ratio (SNR) of an image. Using higher magnification (and a smaller pixel size) slightly enhances the SNR for a given detector detective quantum efficiency, which is crucial to resolve small proteins. However, the reduced field of view at higher magnification captures fewer particles per image, which necessitates the generation of proportionally larger datasets, longer total acquisition times, and greater costs. Because the imaged area decreases quadratically with pixel size, a reduction in the pixel size from 1.0 to 0.5 Å results in a fourfold smaller field of view, which requires an equivalent increase in acquisition speed to maintain efficiency. Under a constant total electron dose (e⁻/Å²) and a constant dose rate (e⁻/physical pixel/s), the exposure time required per image is proportional to the pixel area. Hence, the exposure time can be proportionally reduced (e.g., from 2 s at 1.0 Å/pix to 0.5 s at 0.5 Å/pix). In practice, this proportional reduction in exposure time does not translate into proportional gains in throughput owing to camera-induced delays. As shown in Supplementary Table 1, a reduction in the exposure time from 2 s to 0.5 s decreases the total acquisition time by a factor of 1.46 when acquiring 100 targets using conventional BIS (K3 direct electron detector, 0.1 s/frame). Under identical conditions, CR-BIS achieves a larger improvement (factor of 2.59). These results demonstrate that CR-BIS substantially improves high-magnification throughput compared with conventional BIS, which approaches the ideal scaling in a delay-free acquisition system and facilitates rapid collection of large, high-quality datasets for near-atomic resolution analysis.

### Application to single-particle cryo-EM and cryo-ET

**Fig. 3:**
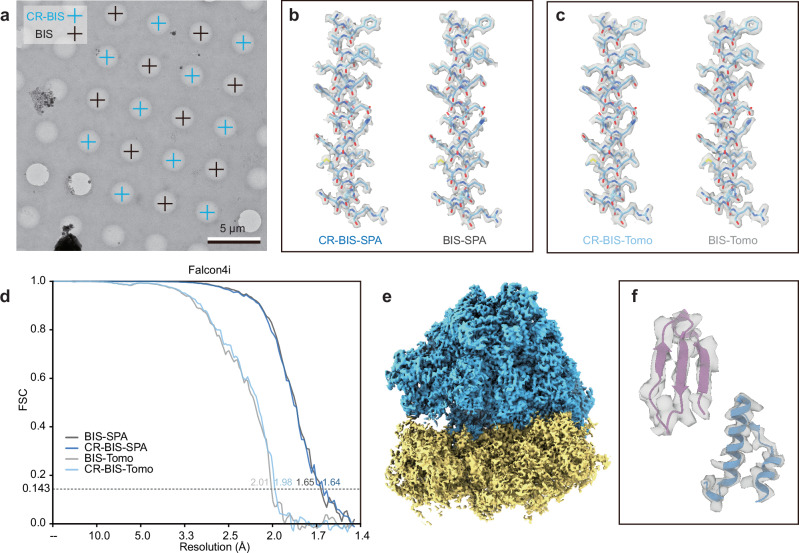
Evaluation of data quality using CR-BIS. **a** Schematic of the interleaved acquisition strategy used to minimize spatial heterogeneity across the grid. Black crosses indicate BIS acquisition positions, and blue crosses indicate CR-BIS acquisition positions. Scale bar = 5 µm. **b** Representative helical density from single-particle reconstructions of apo-ferritin collected on the Falcon 4i direct electron detector using BIS-SPA and CR-BIS-SPA, with the atomic model (Protein Data Bank accession 6Z9E^35^) fitted. Both reconstructions were generated from ~81.7k particles at a pixel size of 0.73 Å and reached comparable resolutions. **c** Representative helical density from subtomogram averaging reconstructions of apo-ferritin collected on the Falcon 4i direct electron detector using BIS-Tomo and CR-BIS-Tomo, with the same atomic model (Protein Data Bank accession 6Z9E^35^) fitted. Both reconstructions were generated from ~18.3k particles at a pixel size of 0.73 Å and reached comparable resolutions. **d** Fourier shell correlation (FSC) curves for apo-ferritin SPA reconstructions acquired using BIS (dark gray) and CR-BIS (dark blue), and for cryo-ET reconstructions acquired using BIS (light gray) and CR-BIS (light blue) on the Falcon 4i direct electron detector. **e** Subtomogram average map of the *Saccharomyces cerevisiae* ribosome, locally refined with a large-subunit mask, from ~32.5k particles at a pixel size of 1.57 Å. **f** Representative densities of an α-helix (blue) and a β-sheet (purple) from the ribosomal large subunit, with the atomic model fitted (Protein Data Bank accession 8XU8^36^).

To evaluate whether CR-BIS affects data quality, we collected SPA and cryo-ET datasets using Falcon 4i, Falcon 4, and K3 direct electron detectors. Representative micrographs split from CR-BIS datasets are shown in Supplementary Fig. 4. To minimize potential bias arising from spatial heterogeneity across the grid, BIS and CR-BIS data were acquired using an interleaved targeting strategy (Fig. 3a), where black crosses denote BIS acquisition positions and blue crosses denote CR-BIS acquisition positions.

For single-particle analysis, apo-ferritin datasets were collected on the Falcon 4i direct electron detector using BIS-SPA and CR-BIS-SPA, which yielded 324 micrographs for each condition. After three-dimensional classification, identical numbers of particles (81,738) were selected from both datasets, which resulted in final reconstructions at resolutions of 1.65 Å (BIS-SPA) and 1.64 Å (CR-BIS-SPA). The reconstructed density maps are shown in Fig. 3b, with BIS-SPA displayed in dark gray and CR-BIS-SPA in dark blue. For cryo-ET, apo-ferritin tilt-series datasets were acquired on the Falcon 4i direct electron detector using BIS-Tomo and CR-BIS-Tomo, with 53 tilt series collected for each condition. The datasets were processed using a Warp–Relion-M subtomogram averaging pipeline. After three-dimensional classification, identical numbers of particles (18,252) were retained, which yielded reconstructions at resolutions of 2.01 Å (BIS-Tomo) and 1.98 Å (CR-BIS-Tomo). The corresponding density maps are shown in Fig. 3c, with BIS-Tomo in light gray and CR-BIS-Tomo in light blue. The FSC curves for the Falcon 4i direct electron detector SPA and cryo-ET reconstructions are shown in Fig. 3d. Equivalent comparisons performed on Falcon 4 and K3 direct electron detectors are shown in Supplementary Fig. 5. Across all tested cameras and acquisition modes, the resolutions obtained using BIS and CR-BIS were comparable, which indicates that the substantial efficiency gains of CR-BIS do not compromise final data quality.

In addition, 10 yeast lamellae were prepared by cryo-focused ion beam milling for in situ cryo-ET, and data were collected using CR-BIS-Tomo on a Falcon 4 direct electron detector at a pixel size of 1.57 Å. A total of 166 tilt series were processed using the Warp–Relion-M subtomogram averaging pipeline, which resulted in the reconstruction of the ribosomal large subunit from 32,527 particles at 3.57 Å resolution (Fig. 3e). The representative densities that correspond to α-helices and β-sheets are shown in Fig. 3f. The resolution achieved in this study is comparable to previous in situ cryo-ET studies using similar microscope configurations and conventional acquisition modes, which further demonstrates that CR-BIS substantially improves acquisition efficiency and does not compromise data quality.

### Accuracy performance of CR-BIS in Cryo-ET

**Fig. 4:**
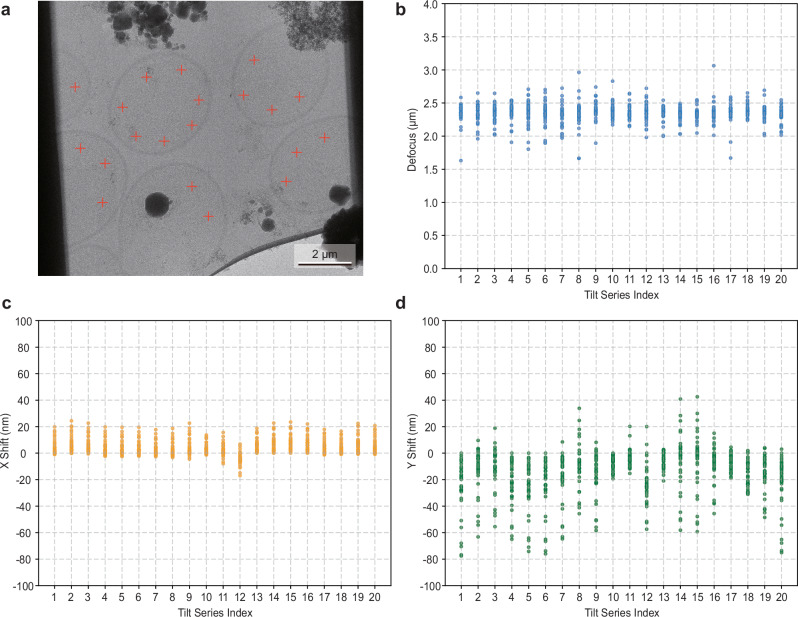
Performance of CR-BIS in Cryo-ET. **a** Representative montage map of a yeast cell lamella (*n* = 10 lamellae) prepared by cryo-focused ion beam (cryo-FIB), with CR-BIS-Tomo target positions indicated by red crosses. Scale bar = 2 µm. **b** Defocus variation across tilt angles for multiple tilt series acquired using CR-BIS-Tomo. Data are shown for 20 tilt series from one focus group. The *x* axis represents the tilt series index, and each cluster of points corresponds to one tilt series. Individual data points within each cluster represent defocus values estimated for different tilt angles within that tilt series, as determined by CTFFIND4^28^. **c**, **d** Specimen shift ranges measured across tilt angles for each tilt series acquired using CR-BIS-Tomo. The *x* axis represents the tilt series index, and individual points correspond to measured *x* direction (**c**) and *y* direction (**d**) shifts for different tilt angles within the same tilt series. The *x*- and *y* directions are defined as parallel and perpendicular to the tilt axis, respectively. Shifts were measured using AreTomo^30^.

Beyond the final reconstruction resolution, the precision of stage tracking during tilt series acquisition is another critical determinant of success in cryo-ET. Poor tracking can introduce significant specimen shifts between consecutive tilt angles, which manifest as translational misalignments in the acquired tilt stack and ultimately lead to limited resolution in subtomogram averaging. Therefore, we evaluated the tracking accuracy of CR-BIS on yeast lamellae samples, where all target positions were collected within a single focusing group (Fig. 4a). The defocus variation within a tilt series was maintained within ±0.5 µm (Fig. 4b), while specimen shifts were constrained within ±20 nm (*x* axis) and ±80 nm (*y* axis) (Fig. 4c, d). This performance is more accurate than the fast-incremental single-exposure scheme (with typically shifts of <500 nm)^24,25^ and is comparable to established BIS-Tomo methods like Pace-Tomo^16^. Shifts in the *y* direction only exceeded 50 nm at high tilt angles (> ±50°). However, the area of interest typically remains within the field of view at these extremes since the imaged area expands by ~30% beyond ±50°. Furthermore, our standard BIS scheme demonstrated excellent alignment, with a *y* shift range of less than ±40 nm (Supplementary Fig. 6). For non-milled, grid-based samples that CR-BIS generally exhibits greater stability, the defocus variation and *x*/*y* shifts were further reduced, as detailed in Supplementary Fig. 7.

In summary, CR-BIS overcomes acquisition delays imposed by the camera and significantly accelerates cryo-EM data collection. The performance gains and preservation of data quality afforded by CR-BIS offers a robust, scalable solution for high-throughput SPA and cryo-ET acquisition. We anticipate that CR-BIS will be particularly valuable for high-throughput structural biology where speed and data quality are critical, such as large-scale drug screening, in situ cellular tomography, and time-resolved cryo-EM.

## Discussion

CR-BIS significantly improves cryo-EM data acquisition efficiency through the reduction of camera-induced delays, which enhances the EAR. For SPA, the EAR reached ~90%, which indicates that there is little room for further improvement. The remaining inefficiencies arise from three main sources described below. First, per-group focusing operations introduce a fixed overhead that becomes increasingly dominant at short exposure times. An acceleration of auto-focusing could further reduce this contribution. Second, beam-blanking, beam-unblanking, and beam-image-shift operations introduce a finite inter-target interval that is currently included in the total exposure setting time, and faster hardware or tighter synchronization could substantially reduce this overhead. Third, the increase in exposure time-dependent slope-derived camera delay correlates with longer CR. Addressing this component may require further optimization of the camera firmware or driver.

The incorporation of the CR mode in cryo-ET doubles the EAR. However, stage tilting, focusing, and tracking steps still occupy a large fraction of the total time in tomography, as shown in Fig. 1b. The recently developed fast-incremental single-exposure method effectively reduces stage-settling delays through continuous incremental tilting; however, its lack of intermediate focusing and image-shift alignment introduces residual image shifts, which limits its practical application^24,25^. To further enhance EAR in cryo-ET, it will be critical to stabilize the sample stages to reduce the time spent on tracking and focusing. Although CR-BIS represents an important step forward, tomography throughput remains more limited than SPA, which leaves room for methodological improvement.

Quantitative analysis (Fig. 1c) reveals that different detectors and frame rates affect the theoretical acceleration factor of CR-BIS. Frame rate-related delays increase with longer exposure setting times, which reduces the effective benefit of CR-BIS. This limitation highlights a need for advances in camera hardware. Furthermore, CR generates larger file sizes, and the frame rate determines the maximum stable recording time for the K3 direct electron detector. Under our tested conditions, the maximum stable exposure setting time for the K3 direct electron detector is ~220 s at 0.1 s/frame and 90 s at 0.05 s/frame, while acquisition errors may occur beyond this point. For the Falcon 4 direct electron detector (where the EER format fixes the frame rate), the maximum continuous exposure setting time allowed by the hardware was ~60 s, which constrains the maximum number of positions per BIS group. Smaller groups increase the number of focusing events, which limits acquisition efficiency.

Unlike conventional BIS, CR-BIS cannot provide immediate previews after each target exposure owing to its CR design. For users who require real-time monitoring to assess data quality or adjust acquisition parameters, this delay may be inconvenient. For SPA and cryo-ET using CR-BIS, previews requires an additional frame-splitting step, which can only be performed after the completion of an entire group acquisition. This introduces an average lag of 15 s (for CR-BIS-Tomo) to 1 min (for CR-BIS-SPA) based on the T_total_ and target number of each focusing group. Nevertheless, the resulting per-target files are fully compatible with existing preprocessing and data transfer pipelines once splitting is complete. To facilitate adoption, we provide an integrated SerialEM script supporting K3, Falcon 4, and Falcon 4i direct electron detectors across multiple modes. The script enables a straightforward switch between conventional BIS (BIS-SPA and BIS-Tomo) and CR-BIS modes through parameter adjustments. For rapid sample screening and feedback, users may still employ conventional BIS, and transition to CR-BIS for high-throughput collection once sample quality is confirmed.

## Methods

### Sample preparation

Single-particle sample: Apo-ferritin was diluted to 2 mg/mL, and 3 µL was applied to glow-discharged NiTi R1.2/1.3, 300-mesh Au grids. Grids were blotted for 3 s at 16 °C and 99% humidity, then plunge-frozen in liquid ethane using a Vitrobot (Thermo Fisher Scientific).

Yeast cryo-lamellae: *Saccharomyces cerevisiae* strain BY4741 was cultured in YPD medium at 29 °C overnight until reaching an OD_600_ of 0.6–0.8. Cells were collected, resuspended in fresh medium, and adjusted to an OD_600_ of 2. A 3 µL aliquot of the suspension was applied to glow-discharged copper Quantifoil 200 R1.2/1.3 grids placed in a horizontal orientation. After a 30 s incubation to promote even distribution, the grids were plunge-frozen in liquid ethane using a Leica EM GP (Leica Microsystems) with 4–6 s of back-side blotting at 16 °C and 70% chamber humidity.

#### Cryo-FIB lamella preparation

Cryo-lamellae were produced using an Aquilos-2 FIB-SEM (Thermo Fisher Scientific). Prior to milling, grids were sputter-coated with metallic platinum for 15 s at 30 mA, followed by a 45 s deposition of organo-platinum using the gas-injection system (GIS), and an additional 15 s platinum sputter coat under the same conditions. Milling was performed with a 30 keV Ga⁺ ion beam in multiple steps: coarse milling to ~2 µm thickness with a 0.3 nA beam, thinning to ~500 nm using 0.1 nA, to ~300 nm with 50 pA, to ~200 nm with 30 pA, and finally polishing to ~150–200 nm using a 10 pA ion beam.

#### Calibration before CR-BIS

We recommend calibrating each microscope before adopting CR-BIS. Calibration should include: (i) determining the relationship between exposure setting time and total camera recording times (with particular attention to frame-rate–dependent offsets on the K3 direct electron detector), which allows estimation of the theoretical efficiency gain; (ii) measuring beam blanking and BIS interval times, enabling more accurate calculation of total exposure setting time; and (iii) testing the optimal frame-splitting threshold for reliable downstream processing. These steps ensure robust application of CR-BIS and minimize the risk of delays caused by incomplete frame capture.

To determine the effective frame rate and delay contributions, we recorded a series of exposures with varying exposure setting times and measured the corresponding total record time. The beam-blanking interval during BIS shifts was optimized by analyzing intensity dips in raw frame stacks. Here, we did pre- calibration for two cryo-EM models, Titan Krios 1 equipped K3 direct electron detector (Gatan) (cryo-EM model 1) and Titan Krios G4 equipped Falcon 4 direct electron detector (Thermo Fisher Scientific) (cryo-EM model 2). The blank and BIS interval is 0.22 s and 0.26 s for cryo-EM model 1 and model 2, respectively. To be noticed, there will be delayed response or extra exposure time for different cryo-EM models. For example, for cryo-EM model 1, when switching to the CR-mode in SerialEM, the actual recording by the camera occurs later than the start of the electron beam exposure. Therefore, a certain delay time needs to be added before the exposure begins. The detailed differences in settings for cryo-EM model 1 and 2 can be seen in the script of CR-BIS. These calibrated parameters were then used to establish the optimized total-exposure-time formula and to guide stack segmentation during downstream processing.

For benchmarking speed improvements, control datasets of SPA and tomographic tilt series acquisition were collected using conventional beam-image shift (BIS)^5^, where target positions are switched without stage movement. The speed comparisons of BIS and CR-BIS are summarized in the Table1 and Supplementary Table1. Target positions within each focus group were selected in SerialEM^6,7^. Each group typically contained 10-25 positions arranged within a radius of ~10 µm field of view. Focus was acquired once at the center of each group before imaging the surrounding targets with BIS.

### Apo-ferritin data collection

Apo-ferritin was used as a standard sample to evaluate data acquisition performance across different microscope models using identical collection parameters operating at 300 keV. The three microscope models employed were: a Titan Krios G4 equipped with a Falcon 4i detector, a Titan Krios G4 equipped with a Falcon 4 detector, and a Titan Krios 1 equipped with a K3 detector. Both SPA and cryo-ET datasets were acquired using conventional BIS and CR-BIS modes.

In CR-BIS mode, following an initial focusing step, the camera continuously recorded frames across all target positions within a beam-image-shift group. The electron beam was blanked during beam shifts to minimize unnecessary exposure. In conventional BIS mode, each target position was recorded as an independent movie stack.

For the Falcon 4i SPA dataset, images were acquired at a nominal magnification of 165,000×, corresponding to a calibrated pixel size of 0.73 Å/pixel. The total dose was 30 e⁻/Å², with an exposure time of 1.94 s per target and an exposure rate of 8.21 e⁻/pixel/s. The target defocus range was set to −0.5 to −0.8 μm. A total of 324 micrographs were collected in each acquisition mode (BIS and CR-BIS). Detailed acquisition parameters for the Falcon 4 and K3 detectors are provided in Supplementary Table 2.

For the Falcon 4i cryo-ET dataset, images were acquired at a nominal magnification of 165,000×, corresponding to a calibrated pixel size of 0.73 Å/pixel. A dose-symmetric tilt scheme was employed with a 2° increment over a tilt range of −38° to +38°. The dose per target per tilt was 3.9 e⁻/Å², with an exposure time of 0.31 s per target and an exposure rate of 6.68 e⁻/pixel/s, resulting in a cumulative dose of 152.1 e⁻/Å² per tilt series. The target defocus range was set to −1.5 to −2.5 μm. A total of 53 tilt series were collected in each acquisition mode. Detailed acquisition parameters for the Falcon 4 and K3 detectors are provided in Supplementary Table 3.

### In situ cryo-ET data collection

Yeast lamellae cryo-ET dataset was collected on a Titan Krios G4 microscope operating at 300 keV. Data were acquired using Falcon 4 and tilt series were recorded at a nominal magnification of 81,000×, corresponding to a pixel size of 1.57 Å/pixel. The dose per target per tilt was 3.0 e⁻/Å², with an exposure time of 0.87 s per target. Normal BIS was applied at the first five tilt angles, while CR-BIS was applied for the remaining tilt angles, with each tilt acquired as a single movie stack. A standard dose-symmetric scheme was used with an angular range of ±60° and 3° increments, resulting in a total dose of ~123 e⁻/Å². Detailed acquisition parameters for ribosome datasets are provided in Supplementary Table 3.

### Preprocessing of CR-BIS output

Raw movie stacks were segmented into sub-stacks corresponding to individual acquisition targets by identifying low-intensity beam-blanking frames (Supplementary Fig. 3). For tomographic data, the first five tilts (normal BIS) and the remaining tilts (CR-BIS) were combined into unified tilt-series stacks. Custom Python scripts were used to generate the required.mdoc files, and individual tilt images were assembled into tilt series using either in-house Python scripts or Warp^26^.

### Apo-ferritin SPA data processing

Motion correction and dose-weighting were performed with MotionCor2^27^. CTF parameters were estimated using CTFFIND4^28^. Particles were auto-picked in RELION 3^29^ using a template-based approach and extracted at a binning factor of 4× (2D classification) and 2× (3D classification). Particles are re-extracted at unbinned pixel size for auto-refinement and iterative CTF refinement were carried out in RELION3^29^. Detailed data processing parameters for apo-ferritin datasets are provided in Supplementary Table 2 and Supplementary Fig. 8. Comprehensive local resolution and angular distribution data for each camera and acquisition method are summarized in Supplementary Tables 4–6.

### Subtomogram averaging of apo-ferritin and ribosome

Both apo-ferritin and ribosome datasets were processed using the Warp–Relion-M pipeline for cryo-ET data. Motion correction and CTF estimation were performed in Warp^26^. Tilt-series alignment was carried out in AreTomo^30^, and the alignment parameters were imported into Warp^26^ for tomogram reconstruction at 4×. Particles were identified via PyTom^31^ template matching using a low-resolution reference. The detailed processing workflow for the ribosome dataset is illustrated in Supplementary Fig. 9. Sub tilt series were extracted in Warp at a box size of 140 voxels, with an initial pixel size of 3.14 Å. Initial alignment and 3D classification were performed in RELION 5^32^. The final subtomogram average of 32,527 particles was refined using M^33^ with orientation and defocus optimization. Final map resolution reached 3.57 Å, determined by gold-standard FSC (0.143 criterion) using a soft mask. The apo-ferritin dataset was processed following an identical workflow, with detailed parameters provided in Supplementary Table 3. Comprehensive local resolution and angular distribution data for each camera and acquisition method are summarized in Supplementary Tables 4–6.

### Reporting summary

Further information on research design is available in the Nature Portfolio Reporting Summary linked to this article.

## Supplementary information

Supplementary Information file

Reporting Summary

Transparent Peer Review file

## Data Availability

The cryo-EM density maps generated in this study have been deposited in the Electron Microscopy Data Bank (EMDB) under the following accession codes: EMD-68810 (BIS-SPA, Falcon4i), EMD-68809 (CR-BIS-SPA, Falcon4i), EMD-68808 (BIS-SPA, Falcon4), EMD-68807 (CR-BIS-SPA, Falcon4), EMD-68812 (BIS-SPA, K3), EMD-68811 (CR-BIS-SPA, K3), EMD-68816 (BIS-Tomo, Falcon4i), EMD-68815 (CR-BIS-Tomo, Falcon4i), EMD-68818 (BIS-Tomo, Falcon4), EMD-68817 (CR-BIS-Tomo, Falcon4), EMD-68814 (BIS-Tomo, K3), EMD-68813 (CR-BIS-Tomo, K3), respectively. The cryo-EM density map of the 60S ribosome has been deposited under accession code EMD-65963 (CR-BIS-Tomo, Falcon4). The raw stack datasets of ferritin collected using CR-BIS-SPA and CR-BIS-Tomo are accessible on EMPIAR under accession codes EMPIAR-13493 and EMPIAR-13494, respectively. All atomic models used in the figures were retrieved from the Protein Data Bank (PDB) with accession codes 6Z9E, 8XU8. Source data for Figs. 1c, 3d, 4b–d and Supplementary Figs. 1, 3, 5c, f, 6, 7, and 9j are provided with the paper.

## Code availability

The software is provided for non-commercial academic research purposes only. Commercial use is strictly prohibited as the underlying method is the subject of a patent (ZL202510925138.0). A custom license specifying these terms is included with the code. All software is available in the GitHub repositories https://github.com/ZHANGXinzheng-LAB/CR-BIS (including SerialEM scripts, a basic tutorial, and Python scripts for preprocessing raw stacks collected by CR-BIS). The version of code used in this study has been deposited in Zenodo: 10.5281/zenodo.17061727^34^.
