# Supplementary material for: Ultra-rapid cryo-EM data acquisition method enabled by continuous recording based beam image shift
Source: Nat Commun. 2026 Jun 12;17:7470. doi: 10.1038/s41467-026-74373-6 (PMC13408471; doi:10.1038/s41467-026-74373-6)
Supplement: Supplementary file 1 — Supplementary Information file [file 41467_2026_74373_MOESM1_ESM.pdf]

Supplementary Information

Ultra-Rapid Cryo-EM Data Acquisition Method Enabled by Continuous Recording Based Beam Image Shift

Qi Yang[1,2†], Xiaojun Huang[3,4,5†], Chunling Wu[1,2], Yan Zeng[3,4,5], Xinzheng Zhang[1,2*]

[1] State Key Laboratory of Biomacromolecules, Institute of Biophysics, Chinese Academy of Sciences, Beijing 100101, China

[2] University of Chinese Academy of Sciences, Beijing, China

[3] Center for Biological Imaging, Core Facilities for Protein Science, Institute of Biophysics, Chinese Academy of Sciences, Beijing, 100101, China

[4] National Multi-mode Trans-scale Biomedical Imaging Center, Beijing, 101408, China

[5] Interdisciplinary Center for Biointelligence, Institute of Biophysics, Chinese Academy of Sciences, Beijing, 101408, China.

[†] These authors contributed equally: Qi Yang, Xiaojun Huang.

[*] Correspondence to: xzzhang@ibp.ac.cn (X.-Z.Z.).

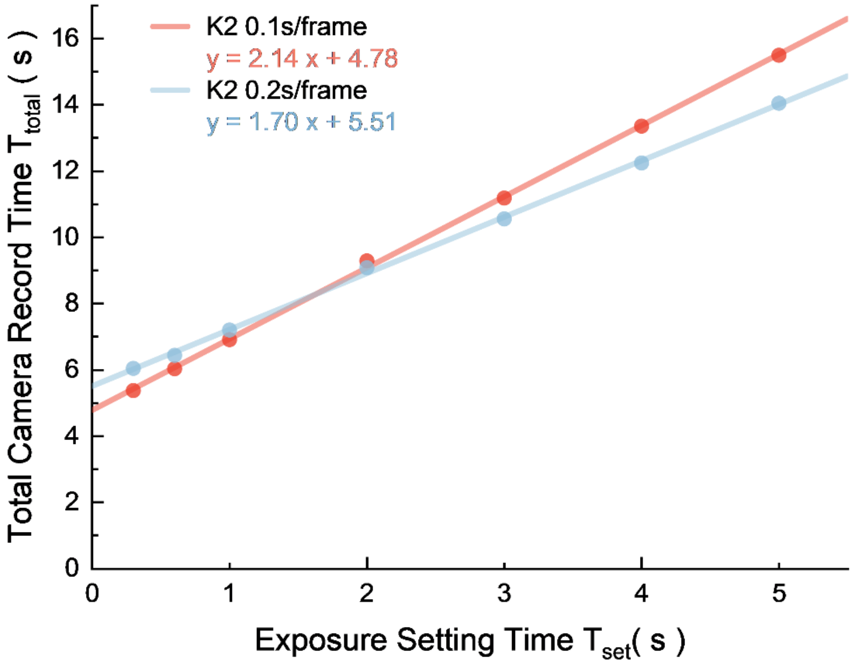

Supplementary Figure 1. The relationship between T_{set} and T_{total} test on K2 camera. Relationship between set exposure time T_{set} and camera total exposure time T_{total} across different frame rates. Each data point is the average of three replicate measurements (n=3). The variation of actual record time with the change of set exposure time using K2 camera with frame rate 0.1 s/frame and 0.2 s/frame were shown with red line and blue line, respectively.

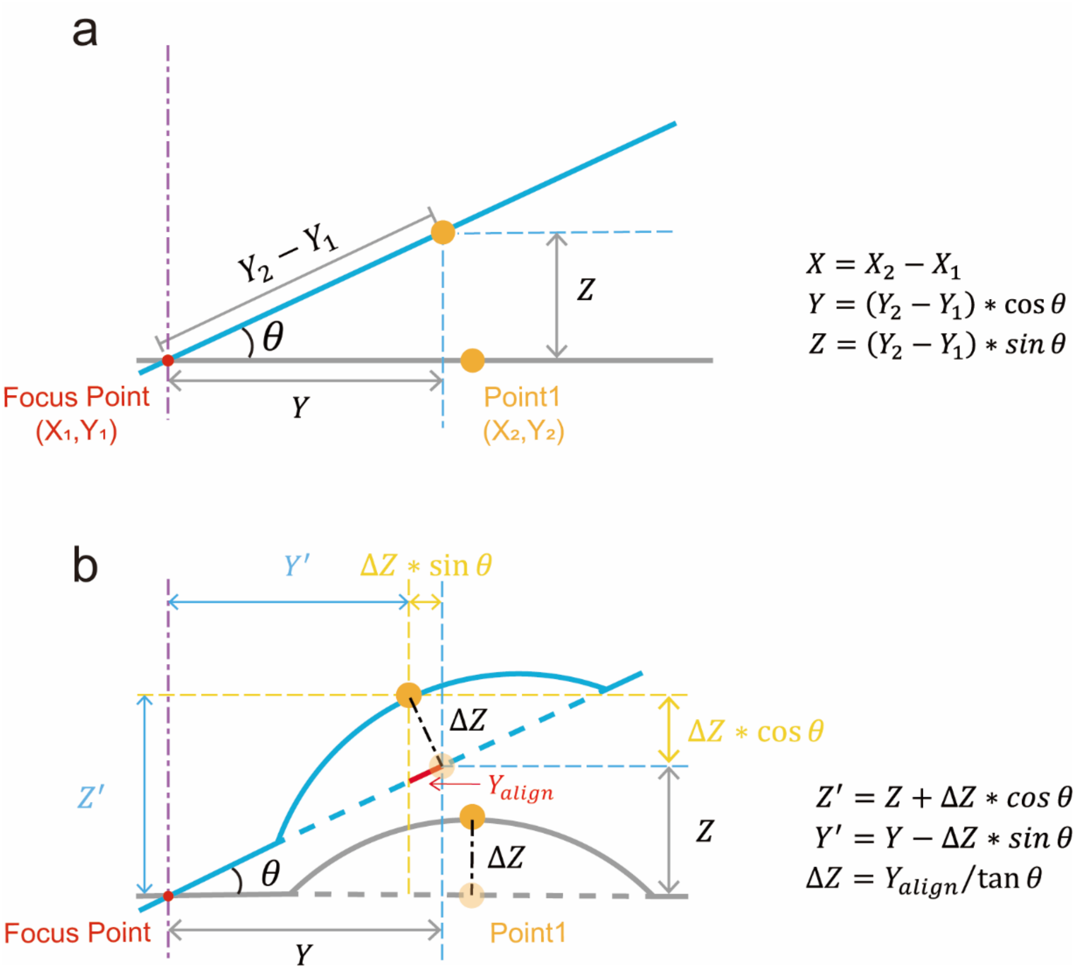

Supplementary Figure 2. Measurement of additional errors caused by uneven sample. a Schematic diagram illustrating the calculation of basic BIS parameters for an ideal sample at a stage tilt angle θ. **b** Schematic diagram showing BIS parameter calculation for real samples with uneven surface. The additional error caused by ΔZ can be derived from the Y_align value provided by tracking after recording, and the BIS parameter is refined for the remaining tilt angles.

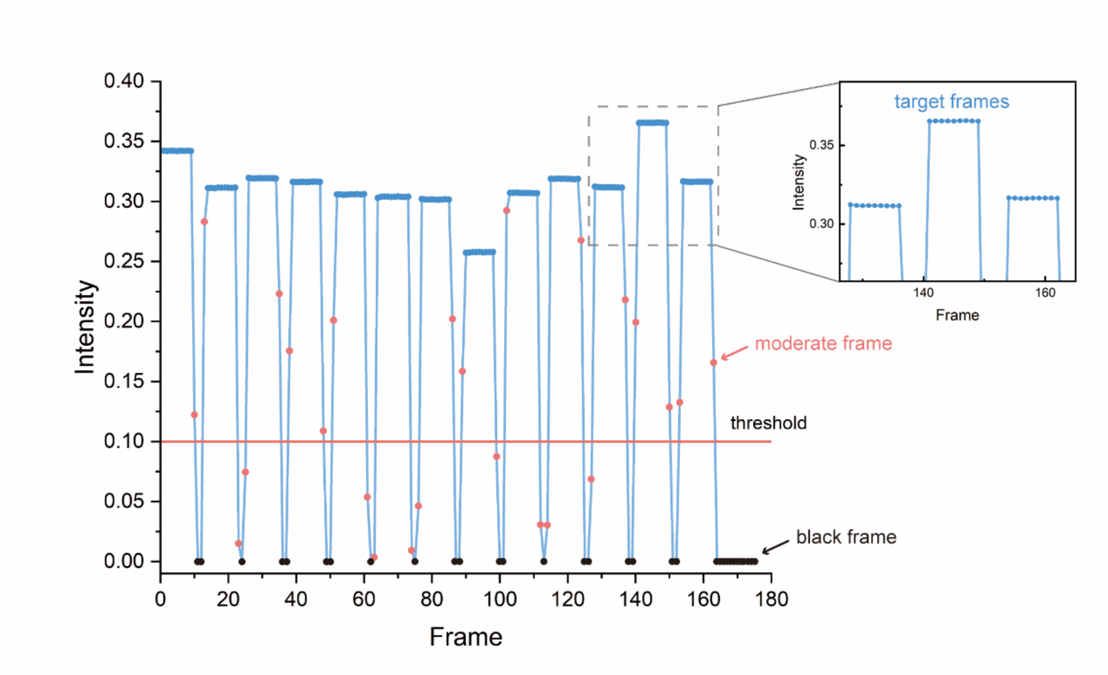

Supplementary Figure 3. Frame intensity identification and raw stack splitting. Target frame intensities (blue dots), beam-blank blank frames (black dots) and moderate frame intensities (red dots) are plotted with an example cutoff threshold (red line, 0.1). The x-axis denotes the frame index, while the y-axis denotes the corresponding frame intensity. Homemade python script was used to analyze the intensity of each frame. A cutoff threshold was applied, and frames with intensities below the threshold were identified as blank frames and removed from the output. A close-up view shows the number of frames in each output file.

	Detector	Set Exposure Time Per point (s)	Frame rate (s/frame)	Point number Per Group	BIS	CR-BIS	Improvement Ratio
SPA	K3	0.5	0.05	25	121 s	35 s	**3.46**
		1	0.05	25	144 s	49 s	**2.94**
		2	0.05	25	192 s	93 s	**2.06**
		0.5	0.1	100	375 s	98 s	**3.83**
		1	0.1	100	436 s	150 s	**2.91**
		2	0.1	100	547 s	254 s	**2.15**
	Falcon 4	0.5	-	25 *4 groups	411	176 s	**2.33**
		1	-	25 *4 groups	459 s	237 s	**1.93**
		2	-	25 *4 groups	566 s	368 s	**1.53**
Cryo-ET	K3	0.5	0.1	25	3.25 min/ts	1.64 min/ts	**1.98**
		1	0.1	25	3.62 min/ts	1.96 min/ts	**1.87**
		0.5	0.05	25	3.48 min/ts	1.72 min/ts	**2.02**
		1	0.05	25	4.12 min/ts	2.32 min/ts	**1.78**
	Falcon 4	0.5	-	25	2.76 min/ts	1.34 min/ts	**2.05**
		1	-	25	3.14 min/ts	1.77 min/ts	**1.77**

Supplementary Table 1: Collection parameters of speed tests dataset collected using our normal BIS and CR-BIS.

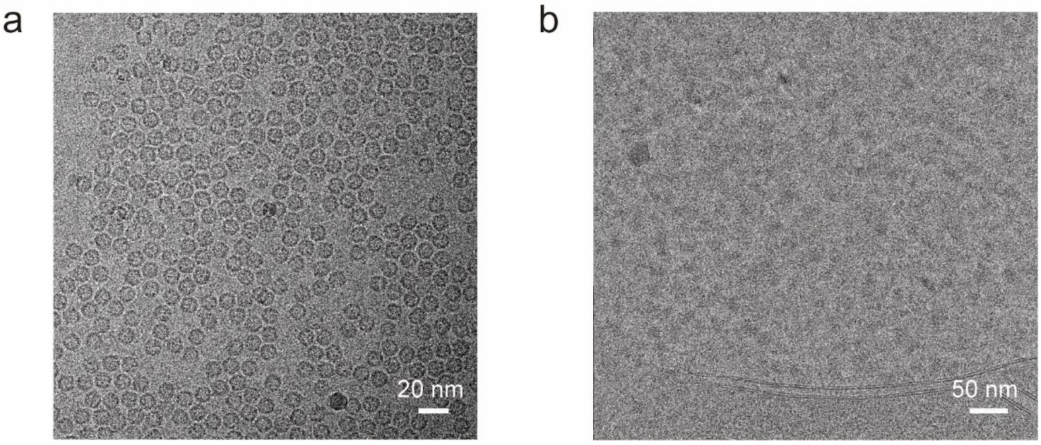

Supplementary Figure 4. Split micrographs from CR-BIS.
a A typical micrograph of CR-BIS-SPA data of apo-ferritin (n = 324 micrographs, scale bar = 20 nm). **b** A typical 0° micrograph of CR-BIS-Tomo data of yeast lamellae (n = 166 tilt series, scale bar = 50 nm).

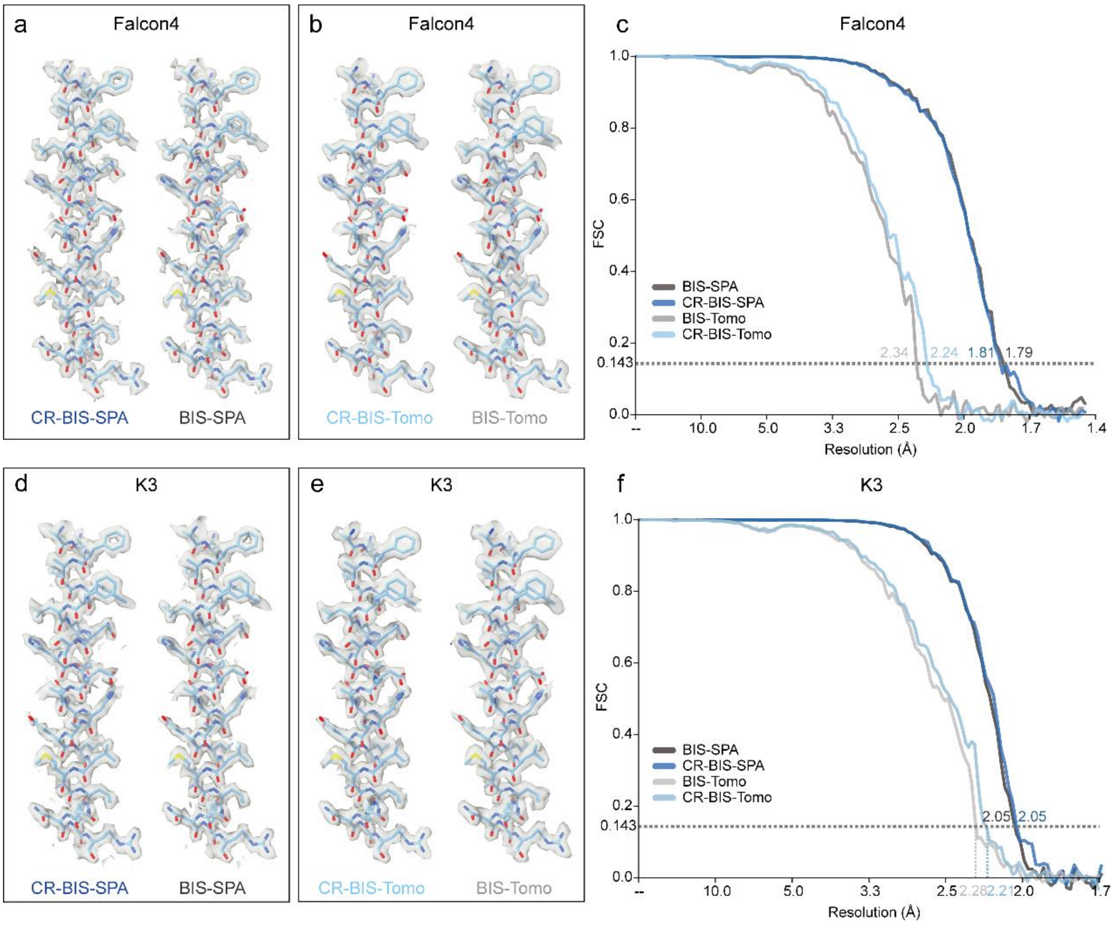

Supplementary Figure 5. Evaluation of data quality using CR-BIS on Falcon 4 and K3 direct electron detector. a Representative helical density from single-particle reconstructions of apo-ferritin collected on the Falcon 4 direct electron detector using BIS-SPA and CR-BIS-SPA, with the atomic model (Protein Data Bank accession 6Z9E fitted. Both reconstructions were generated from 46,217 particles and reached comparable resolutions. **b** Representative helical density from subtomogram averaging reconstructions of apo-ferritin collected on the Falcon 4 direct electron detector using BIS-Tomo and CR-BIS-Tomo, with the same atomic model (Protein Data Bank accession 6Z9E) fitted. Both reconstructions were generated from 13,013 particles and reached comparable resolutions. **c** Fourier shell correlation (FSC) curves for apo-ferritin SPA reconstructions acquired using BIS (dark grey) and CR-BIS (dark blue), and for cryo-ET reconstructions acquired using BIS (light grey) and CR-BIS (light blue) on the Falcon 4 direct electron detector. **d** Representative helical density from single-particle reconstructions of apo-ferritin collected on the K3 direct electron detector using BIS-SPA and CR-BIS-SPA, with the atomic model (Protein Data Bank accession 6Z9E) fitted. Both reconstructions were generated from 172,347 particles and reached comparable resolutions. **e** Representative helical density from subtomogram averaging reconstructions of apo-ferritin collected on the K3 direct electron detector using BIS-Tomo and CR-BIS-Tomo, with the same atomic model (Protein Data Bank accession 6Z9E) fitted. Both reconstructions were generated from 17,062 particles and reached comparable resolutions. **f** FSC curves for apo-ferritin SPA reconstructions acquired using BIS (dark grey) and CR-BIS (dark blue), and for cryo-ET reconstructions acquired using BIS (light grey) and CR-BIS (light blue) on the K3 direct electron detector.

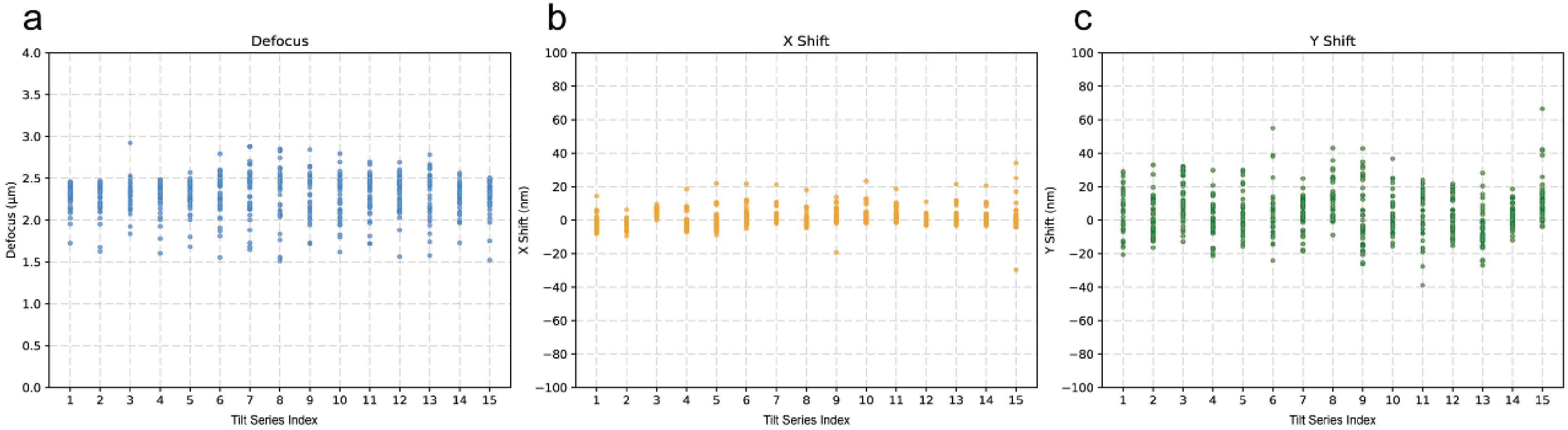

Supplementary Figure 6. The tilt alignment parameter of lamellae-sample collected by normal BIS.
a Defocus variation across tilt angles for multiple tilt series acquired using BIS-Tomo. Data are shown for 15 tilt series from one focus group. The x-axis represents the tilt series index, and each cluster of points corresponds to one tilt series. Individual data points within each cluster represent defocus values estimated for different tilt angles within that tilt series, as determined by CTFFIND4. **b c** Specimen shift ranges measured across tilt angles for each tilt series acquired using BIS-Tomo. The x-axis represents the tilt series index, and individual points correspond to measured x-direction **(b)** and y-direction **(c)** shifts for different tilt angles within the same tilt series. The x- and y-directions are defined as parallel and perpendicular to the tilt axis, respectively. Shifts were measured using AreTomo3.

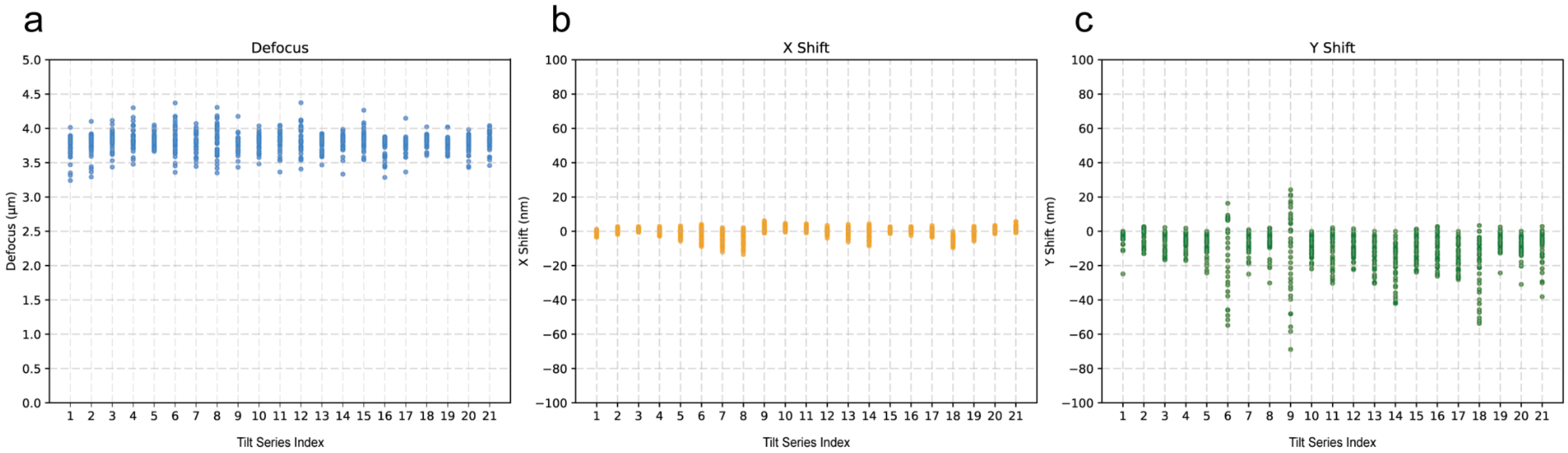

Supplementary Figure 7. The tilt alignment parameter of grid-sample collected by CR-BIS.
a Defocus variation across tilt angles for multiple tilt series acquired using CR-BIS-Tomo. Data are shown for 21 tilt series from one focus group. The x-axis represents the tilt series index, and each cluster of points corresponds to one tilt series. Individual data points within each cluster represent defocus values estimated for different tilt angles within that tilt series, as determined by CTFFIND4. **b c** Specimen shift ranges measured across tilt angles for each tilt series acquired using CR-BIS-Tomo. The x-axis represents the tilt series index, and individual points correspond to measured x-direction **b** and y-direction **c** shifts for different tilt angles within the same tilt series. The x- and y-directions are defined as parallel and perpendicular to the tilt axis, respectively. Shifts were measured using AreTomo3.

Dataset	Single Particle Dataset of Human Apo-ferrtin					
Microscope	Titan Krios G4		Titan Krios G4		Titan Krios 1	
Detector	Falcon 4i		Falcon 4		K3	
Voltage (kV)	300	300	300	300	300	300
Energy filter slit width (eV)	10	10	10	10	-	-
Acquisition mode	BIS	CR-BIS	BIS	CR-BIS	BIS	CR-BIS
Nominal manification (×)	165,000	165,000	165,000	165,000	290,000	290,000
Pixel size (Å/pixel)	0.73	0.73	0.73	0.73	0.83	0.83
Target defocus range (µm)	-0.5 to -0.8	-0.5 to -0.8	-0.8 to -1.2	-0.8 to -1.2	-0.8 to -1.2	-0.8 to -1.2
Total exposure time per target point (s)	1.94	1.94	3.27	3.27	1.50	1.50
Total dose (e^{-}/Å²)	30	30	30	30	30	30
Exposure rate (e^{-}/pixel/s)	8.21	8.21	4.90	4.90	13.78	13.78
Number of micrographs	324	324	290	290	471	471
Initial particles number	118,089	122,535	60,378	59,654	293,164	278,125
Final particles number	81,738	81,738	46,217	46,217	172,347	172,347
Symmetry imposed	O	O	O	O	O	O
Map resolution (Å)	1.65	1.64	1.79	1.81	2.05	2.05
FSC threshold	0.143	0.143	0.143	0.143	0.143	0.143
EMDB	EMD-68810	EMD-68809	EMD-68808	EMD-68807	EMD-68812	EMD-68811
EMPIAR	-	EMPIAR-13493	-	-	-	-

Supplementary Table 2: Collection parameters of SPA datasets.

Dataset	Cryo-ET Dataset						
Sample	Human Apo-ferritin		Human Apo-ferritin		Human Apo-ferritin		Ribosome
Microscope	Titan Krios G4		Titan Krios G4		Titan Krios 1		Titan Krios G4
Detector	Falcon 4i		Falcon 4		K3		Falcon 4
Voltage (kV)	300	300	300	300	300	300	300
Energy filter slit width (eV)	10	10	10	10	-	-	10
Acquisition mode	BIS	CR-BIS	BIS	CR-BIS	BIS	CR-BIS	CR-BIS
Nominal manification (×)	165,000	165,000	165,000	165,000	290,000	290,000	81,000
Pixel size (Å/pixel)	0.73	0.73	0.73	0.73	0.83	0.83	1.57
Target defocus range (µm)	-1.5 to -2.5	-1.5 to -2.5	-1.5 to -2.5	-1.5 to -2.5	-1.5 to -2.5	-1.5 to -2.5	-2.0 to -2.5
Total exposure time per target point per tilt (s)	0.31	0.31	0.25	0.25	0.50	0.50	0.845
Exposure rate (e^-/pixel/s)	6.68	6.68	8.54	8.54	5.51	5.51	8.57
Electron fluence per tilt (e^- /Å^2)	3.9	3.9	4.0	4.0	4.0	4.0	3.0
Milling angle (°)	-	-	-	-	-	-	10
Tilt range (°)	-38 to 38	-38 to 38	-38 to 38	-38 to 38	-38 to 38	-38 to 38	-60 to 60
Tilt increment (°)	2	2	2	2	2	2	3
Total electron fluence (e^- /Å^2)	152.1	152.1	156.0	156.0	156.0	156.0	123.0
Number of tilt series	53	53	56	56	52	52	166
Final particles number	18,252	18,252	13,013	13,013	17,062	17,062	32,527
Symmetry imposed	O	O	O	O	O	O	O
Map resolution (Å)	2.01	1.98	2.34	2.24	2.28	2.21	3.57
FSC threshold	0.143	0.143	0.143	0.143	0.143	0.143	0.143
EMDB	EMD-68816	EMD-68815	EMD-68818	EMD-68817	EMD-68814	EMD-68813	EMD-65963
EMPIAR	-	-	-	EMPIAR-13494	-	-	-

Supplementary Table 3: Collection parameters of cryo-ET datasets.

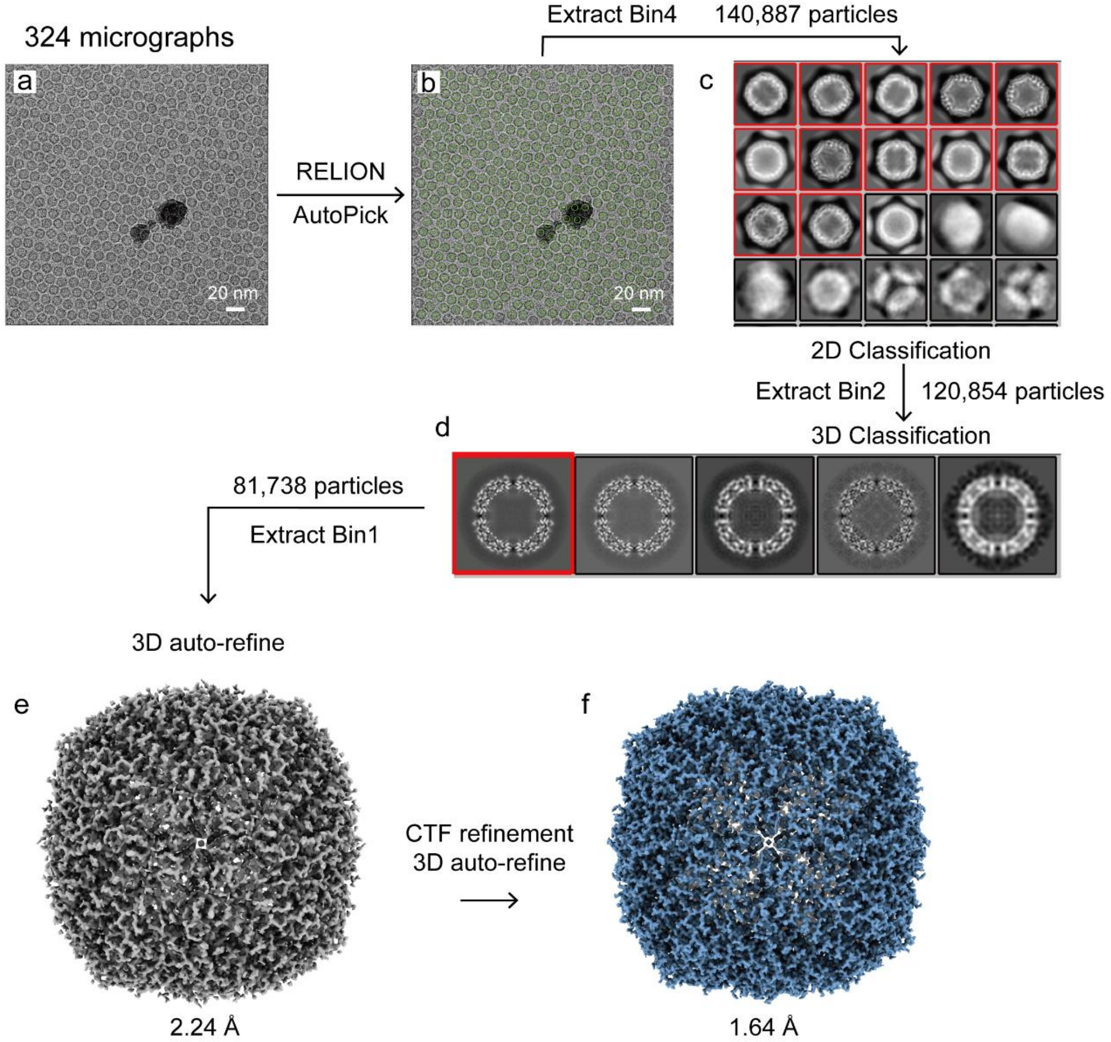

Supplementary Figure 8. Single-particle analysis (SPA) data processing workflow for the apo-ferritin dataset collected using CR-BIS on Falcon 4i direct electron detector. Processing steps are connected by lines. **a** A total of 324 micrographs of apo-ferritin were collected using CR-BIS. b) Example RELION auto-picking results with particle locations indicated (green circles). **c** Representative 2D classification of 140,887 picked particles, with 120,854 particles selected for further processing. **d** Initial 3D classification yielded five classes, from which 81,738 particles (red box) were selected for refinement. **e** The selected 81,738 particles from **d** (red box) were re-extracted for refinement and auto-refinement in RELION produced a map at 2.24 Å resolution. ff) After CTF refinement, the final map of apo-ferritin reached 1.64 Å resolution (gold-standard FSC = 0.143).

Falcon4i	CR-BIS-SPA	BIS-SPA	CR-BIS-Tomo	BIS-Tomo
Final Reconstruction	1.64 Å 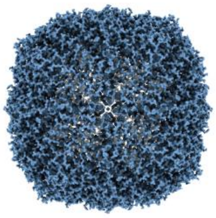	1.65 Å 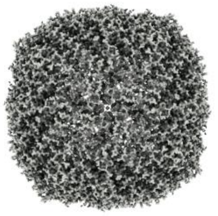	1.98 Å 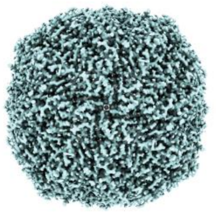	2.01 Å 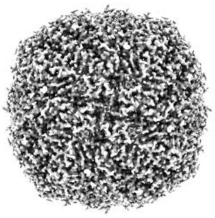
Local Resolution Map	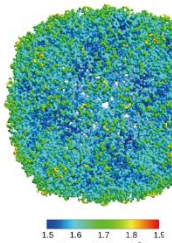	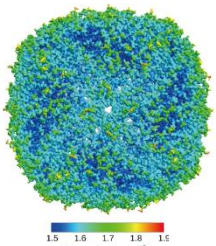	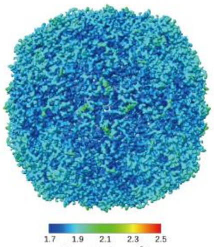	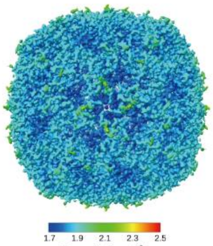
Angular Distribution	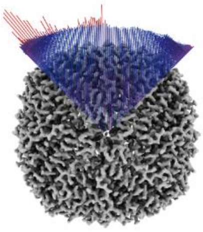	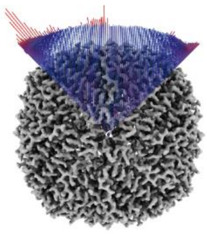	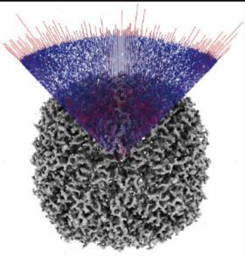	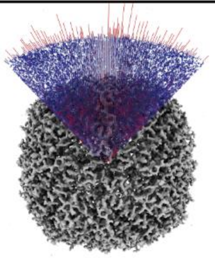

Supplementary Table 4: Comparison of final map, local resolution-colored map, and angular distribution for Falcon4i under different acquisition methods.

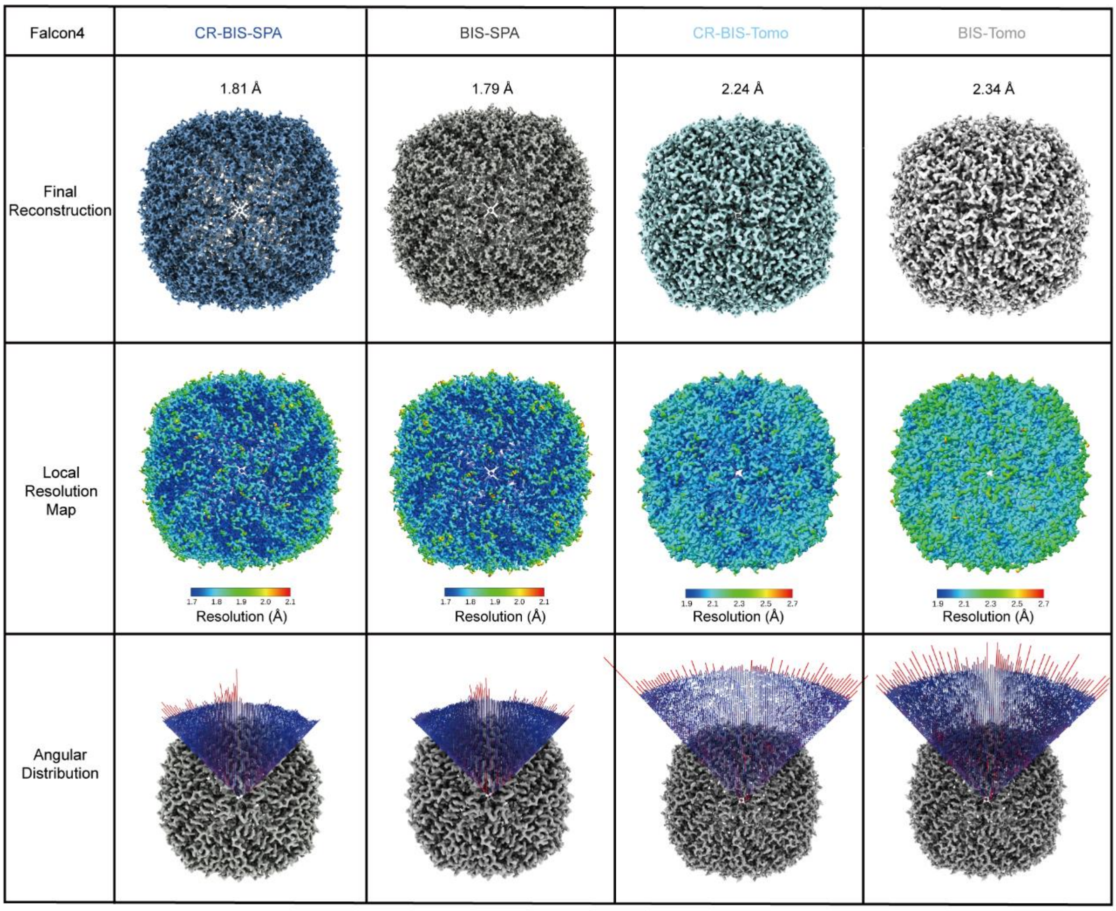

Supplementary Table 5: Comparison of final map, local resolution-colored map, and angular distribution for Falcon4 under different acquisition methods.

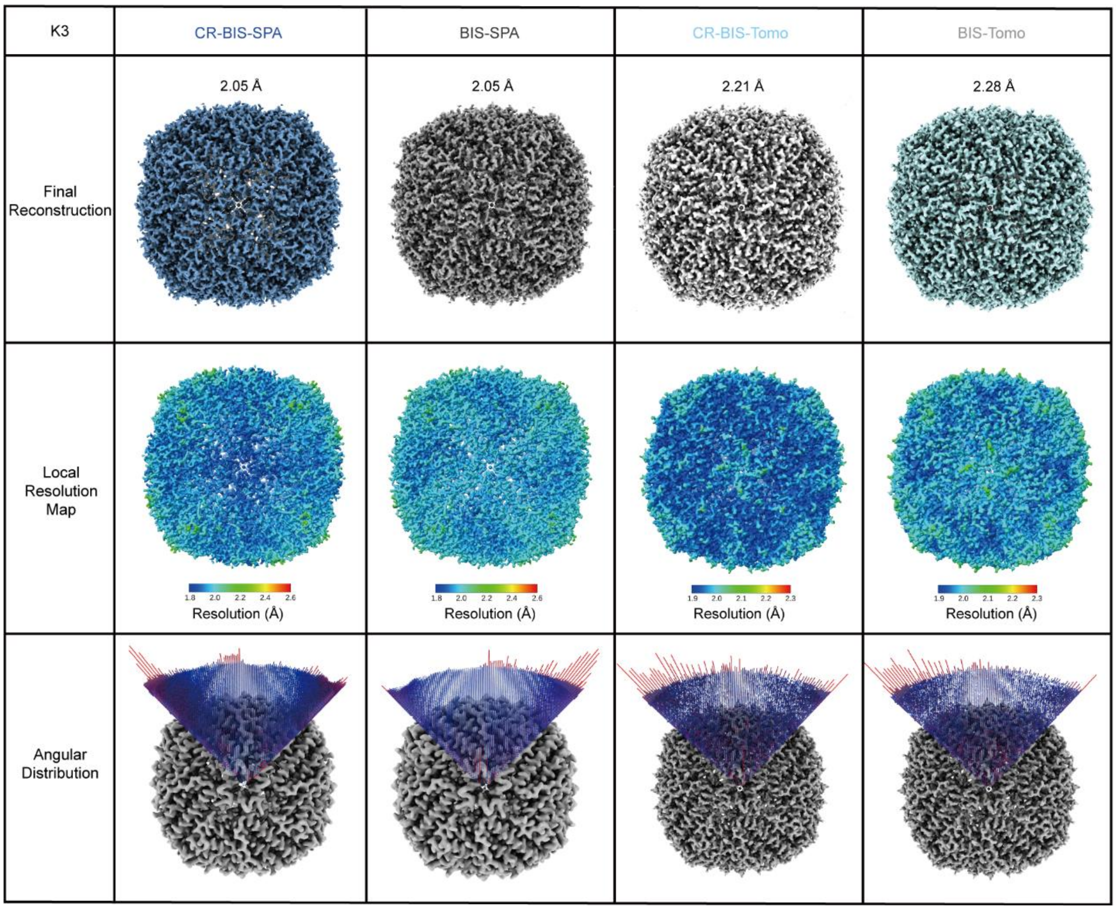

Supplementary Table 6: Comparison of final map, local resolution-colored map, and angular distribution for K3 under different acquisition methods.

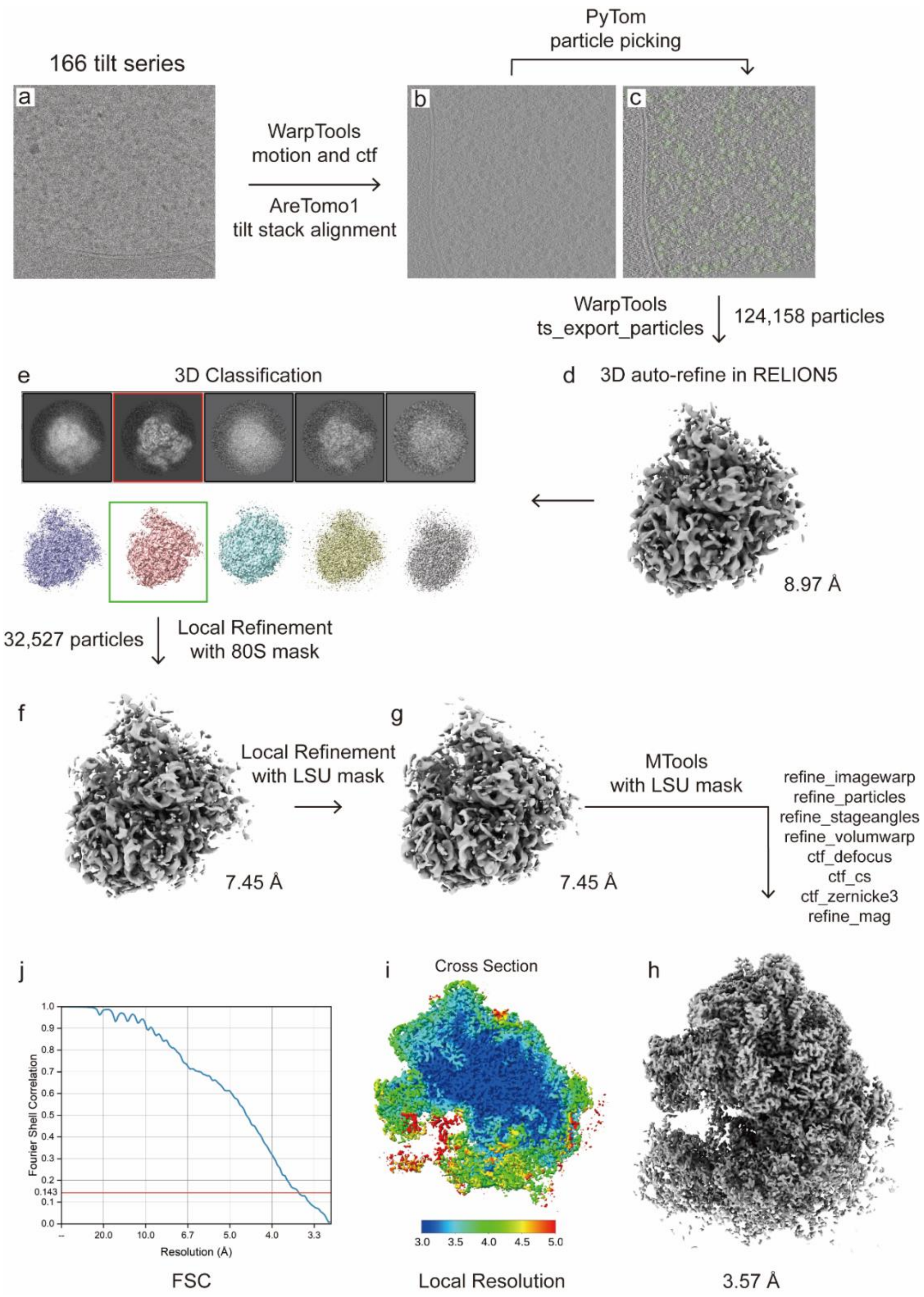

Supplementary Figure 9. Subtomogram averaging (STA) processing workflow for the ribosome dataset collected from yeast lamellae using CR-BIS. Processing steps are connected by lines. **a** 166 tilt series of yeast lamellae were collected using CR-BIS, with motion correction and CTF estimation performed in Warp. **b** Tilt-series alignment was carried out in AreTomo1, followed by tomogram reconstruction in Warp. **c** Ribosomes were identified by PyTom template matching, yielding 124,158 candidate particles. **d** Subtomograms were extracted in Warp and imported into RELION 5 for initial alignment and yielded ribosome density maps at 8.97 Å. **e** After 3D classification, 32,527 particles were retained for local refinement with an 80S mask. **f-g** Local refinements with 80S and LSU masks yielded ribosome density maps at 7.45 Å resolution. h) Final refinement in M produced a ribosome map at 3.57 Å resolution (gold-standard FSC = 0.143). **i** Local resolution map of the LSU. **j** Final FSC curve corresponding to the refined map.
