# Supplementary material for: Ultra-rapid cryo-EM data acquisition method enabled by continuous recording based beam image shift
Source: Nat Commun. 2026 Jun 12;17:7470. doi: 10.1038/s41467-026-74373-6 (PMC13408471; doi:10.1038/s41467-026-74373-6)
Supplement: Supplementary file 2 — Reporting Summary [file 41467_2026_74373_MOESM2_ESM.pdf]

nature portfolio

Corresponding author(s): Xinzheng Zhang

Last updated by author(s): Apr 30, 2026

Reporting Summary

Nature Portfolio wishes to improve the reproducibility of the work that we publish. This form provides structure for consistency and transparency in reporting. For further information on Nature Portfolio policies, see our Editorial Policies and the Editorial Policy Checklist.

Statistics

For all statistical analyses, confirm that the following items are present in the figure legend, table legend, main text, or Methods section.

n/a	Confirmed	
☐	☒	The exact sample size (*n*) for each experimental group/condition, given as a discrete number and unit of measurement
☐	☒	A statement on whether measurements were taken from distinct samples or whether the same sample was measured repeatedly
☒	☐	The statistical test(s) used AND whether they are one- or two-sided<br>*Only common tests should be described solely by name; describe more complex techniques in the Methods section.*
☐	☒	A description of all covariates tested
☒	☐	A description of any assumptions or corrections, such as tests of normality and adjustment for multiple comparisons
☒	☐	A full description of the statistical parameters including central tendency (e.g. means) or other basic estimates (e.g. regression coefficient) AND variation (e.g. standard deviation) or associated estimates of uncertainty (e.g. confidence intervals)
☒	☐	For null hypothesis testing, the test statistic (e.g. *F*, *t*, *r*) with confidence intervals, effect sizes, degrees of freedom and *P* value noted<br>*Give P values as exact values whenever suitable.*
☒	☐	For Bayesian analysis, information on the choice of priors and Markov chain Monte Carlo settings
☒	☐	For hierarchical and complex designs, identification of the appropriate level for tests and full reporting of outcomes
☒	☐	Estimates of effect sizes (e.g. Cohen's *d*, Pearson's *r*), indicating how they were calculated

Our web collection on statistics for biologists contains articles on many of the points above.

Software and code

Policy information about availability of computer code

Data collection	Data were collected using SerialEM (version 4.2) with custom CR-BIS scripts for high-throughput continuous recording acquisition. The CR-BIS scripts are central to this study and have been made available for reviewers and editors (see code availability statement below).
Data analysis	The following software were used for data processing and analysis:<br>Software & Purpose<br>MotionCor2 1.1.4: Motion correction and dose-weighting for SPA data<br>CTFFIND 4.1.14: CTF parameter estimation<br>Warp 2.0(Warp linux version): Motion correction, CTF estimation, and tomogram reconstruction for cryo-ET data<br>AreTomo 1.0: Tilt-series alignment<br>RELION 5.0: particle picking, 2D classification, 3D classification, refinement, local resolution estimate for SPA; 3D classification adn refinement for subtomogram averaging<br>M: Final subtomogram refinement with orientation and defocus optimization, local resolution map for subtomogarm averaging<br>Origin 2025b: Data visualization and figure generation<br>Microsoft Excel: Data organization and basic statistical analysis<br>UCSF ChimeraX 1.6.1: presenting electron density maps

For manuscripts utilizing custom algorithms or software that are central to the research but not yet described in published literature, software must be made available to editors and reviewers. We strongly encourage code deposition in a community repository (e.g. GitHub). See the Nature Portfolio guidelines for submitting code & software for further information.

Data

Policy information about availability of data

All manuscripts must include a data availability statement. This statement should provide the following information, where applicable:

- Accession codes, unique identifiers, or web links for publicly available datasets
- A description of any restrictions on data availability
- For clinical datasets or third party data, please ensure that the statement adheres to our policy

The cryo-EM density maps generated in this study have been deposited in the Electron Microscopy Data Bank (EMDB) under the following accession codes: EMD-68810[https://www.emdataresource.org/EMD-68810] (BIS-SPA, Falcon4i), EMD-68809[https://www.emdataresource.org/EMD-68809] (CR-BIS-SPA, Falcon4i), EMD-68808[https://www.emdataresource.org/EMD-68808] (BIS-SPA, Falcon4), EMD-68807[https://www.emdataresource.org/EMD-68807] (CR-BIS-SPA, Falcon4), EMD-68812[https://www.emdataresource.org/EMD-68812] (BIS-SPA, K3), EMD-68811[https://www.emdataresource.org/EMD-68811] (CR-BIS-SPA, K3), EMD-68816[https://www.emdataresource.org/EMD-68816] (BIS-Tomo, Falcon4i), EMD-68815[https://www.emdataresource.org/EMD-68815] (CR-BIS-Tomo, Falcon4i), EMD-68818[https://www.emdataresource.org/EMD-68818] (BIS-Tomo, Falcon4), EMD-68817[https://www.emdataresource.org/EMD-68817] (CR-BIS-Tomo, Falcon4), EMD-68814[https://www.emdataresource.org/EMD-68814] (BIS-Tomo, K3), EMD-68813[https://www.emdataresource.org/EMD-68813] (CR-BIS-Tomo, K3), respectively. The cryo-EM density map of the 60S ribosome has been deposited under accession code EMD-65963[https://www.emdataresource.org/EMD-65963] (CR-BIS-Tomo, Falcon4). The raw stack datasets of ferritin collected using CR BIS SPA and CR BIS Tomo are accessible on EMPIAR under accession codes EMPIAR-13493 and EMPIAR-13494, respectively. All atomic models used in the figures were retrieved from the Protein Data Bank (PDB) with accession codes 6Z9E[https://www.wwpdb.org/pdb?id=pdb_00006z9e], 8XU852[https://www.wwpdb.org/pdb?id=pdb_00008xu8]. Source data for Figure 1c, 3d, 4bcd and Supplementary Figure 1, 3, 5cf, 6, 7, 9j are provided with the paper.

Research involving human participants, their data, or biological material

Policy information about studies with human participants or human data. See also policy information about sex, gender (identity/presentation), and sexual orientation and race, ethnicity and racism.

Reporting on sex and gender	*Use the terms sex (biological attribute) and gender (shaped by social and cultural circumstances) carefully in order to avoid confusing both terms. Indicate if findings apply to only one sex or gender; describe whether sex and gender were considered in study design; whether sex and/or gender was determined based on self-reporting or assigned and methods used. Provide in the source data disaggregated sex and gender data, where this information has been collected, and if consent has been obtained for sharing of individual-level data; provide overall numbers in this Reporting Summary. Please state if this information has not been collected. Report sex- and gender-based analyses where performed, justify reasons for lack of sex- and gender-based analysis.*
Reporting on race, ethnicity, or other socially relevant groupings	*Please specify the socially constructed or socially relevant categorization variable(s) used in your manuscript and explain why they were used. Please note that such variables should not be used as proxies for other socially constructed/relevant variables (for example, race or ethnicity should not be used as a proxy for socioeconomic status). Provide clear definitions of the relevant terms used, how they were provided (by the participants/respondents, the researchers, or third parties), and the method(s) used to classify people into the different categories (e.g. self-report, census or administrative data, social media data, etc.) Please provide details about how you controlled for confounding variables in your analyses.*
Population characteristics	*Describe the covariate-relevant population characteristics of the human research participants (e.g. age, genotypic information, past and current diagnosis and treatment categories). If you filled out the behavioural & social sciences study design questions and have nothing to add here, write "See above."*
Recruitment	*Describe how participants were recruited. Outline any potential self-selection bias or other biases that may be present and how these are likely to impact results.*
Ethics oversight	*Identify the organization(s) that approved the study protocol.*

Note that full information on the approval of the study protocol must also be provided in the manuscript.

Field-specific reporting

Please select the one below that is the best fit for your research. If you are not sure, read the appropriate sections before making your selection.

☒ Life sciences ☐ Behavioural & social sciences ☐ Ecological, evolutionary & environmental sciences

For a reference copy of the document with all sections, see nature.com/documents/nr-reporting-summary-flat.pdf

Life sciences study design

All studies must disclose on these points even when the disclosure is negative.

Sample size	No formal statistical methods were used to predetermine sample size. Sample sizes were chosen based on standard practices in cryo-EM and cryo-ET data collection, ensuring sufficient particle numbers to achieve high-resolution reconstructions and enable reliable comparison between BIS and CR-BIS acquisition modes.

Data exclusions	No data were excluded from the analyses, except for routine preprocessing steps (e.g., removal of low-quality particles during 2D/3D classification), which are standard in cryo-EM data processing workflows. No dataset-specific exclusion criteria were applied.
Replication	All experiments were reproducible across multiple datasets acquired using different detectors (Falcon 4i, Falcon 4, and Gatan K3) and acquisition modes (BIS and CR-BIS). Comparable reconstruction quality was consistently obtained, confirming the robustness and reproducibility of the CR-BIS method.
Randomization	Single particle data acquisition targets were selected using an interleaved strategy on the same grid and mesh, with BIS and CR-BIS positions alternated to minimize spatial bias. Formal randomization was not required, as all comparisons were performed under matched experimental conditions.
Blinding	Blinding was not relevant to this study, as data acquisition and analysis were performed using automated pipelines without subjective outcome assessment.

Reporting for specific materials, systems and methods

We require information from authors about some types of materials, experimental systems and methods used in many studies. Here, indicate whether each material, system or method listed is relevant to your study. If you are not sure if a list item applies to your research, read the appropriate section before selecting a response.

Materials & experimental systems

n/a	Involved in the study
☒	☐ Antibodies
☒	☐ Eukaryotic cell lines
☒	☐ Palaeontology and archaeology
☒	☐ Animals and other organisms
☒	☐ Clinical data
☒	☐ Dual use research of concern
☒	☐ Plants

Methods

n/a	Involved in the study
☒	☐ ChIP-seq
☒	☐ Flow cytometry
☒	☐ MRI-based neuroimaging

Plants

Seed stocks	*Report on the source of all seed stocks or other plant material used. If applicable, state the seed stock centre and catalogue number. If plant specimens were collected from the field, describe the collection location, date and sampling procedures.*
Novel plant genotypes	*Describe the methods by which all novel plant genotypes were produced. This includes those generated by transgenic approaches, gene editing, chemical/radiation-based mutagenesis and hybridization. For transgenic lines, describe the transformation method, the number of independent lines analyzed and the generation upon which experiments were performed. For gene-edited lines, describe the editor used, the endogenous sequence targeted for editing, the targeting guide RNA sequence (if applicable) and how the editor was applied.*
Authentication	*Describe any authentication procedures for each seed stock used or novel genotype generated. Describe any experiments used to assess the effect of a mutation and, where applicable, how potential secondary effects (e.g. second site T-DNA insertions, mosiacism, off-target gene editing) were examined.*
