# Supplementary material for: Ultra-rapid cryo-EM data acquisition method enabled by continuous recording based beam image shift
Source: Nat Commun. 2026 Jun 12;17:7470. doi: 10.1038/s41467-026-74373-6 (PMC13408471; doi:10.1038/s41467-026-74373-6)
Supplement: Supplementary file 3 — Transparent Peer Review file [file 41467_2026_74373_MOESM3_ESM.pdf]

nature portfolio

# Peer Review File

# Ultra-Rapid Cryo-EM Data Acquisition Method Enabled by Continuous Recording Based Beam Image Shift

Corresponding Author: Professor Xinzheng Zhang

**This file contains all reviewer reports in order by version, followed by all author rebuttals in order by version.**

Version 0:

Reviewer comments:

Reviewer #1

(Remarks to the Author)
Due to the high cost and the limited instrument time of cryo-EM, it remains a crucial objective to enhance data collection efficiency in order to push the resolution of the reconstruction. In this study, the authors present CR-BIS framework which eliminates camera-induced delay time by continuously recording during beam-image shift acquisition. CR-BIS enables a significant increase in the SPA and cryo-ET data collection without compromising the data quality, indicating a robust and scalable solution for high-throughput SPA and cryo-ET acquisition.
Specific comments:
In Fig. 1a, the EAR is defined as the ratio of the total recording time (dash blue area) to the total time (all areas). In Fig. 1b, the EAR is defined as the ratio of the exposure setting time (dark blue area) to the total time (all areas), and this study uses the second way to define EAR. If yes, the authors should clearly state this difference in the main text or the figurer legend.
In Fig. 1c, please clearly label x-axis and y-axis with Tset and Ttotal.
In line 118, “shown as the dash area in Fig1.a”. It should be “Fig. 1a”.
In line 139, Falcon 4i camera internal frame rate is 320 fps, Falcon 4 is 250 fps. The authors cited two references ([12] and [23]) here, but both references used Falcon 4 camera, not Falcon 4i.
In Supplementary Figure S1, it should be K2 camera, not K22 camera.
In line 158-159, “… control stage movement ahead during the slope-derived delay at the end of long-recording …”. In Fig. 2a, the intercept term and slope term are connected between each other in CR-BIS method. Does this method only control stage movement ahead during the slope-derived delay, not including the intercept-derived delay?
In line 166, when using the first five tilts to calculate Δz without CR mode, is this universal for any tilt angle, step size, or any dose per tilt?
In Supplementary Figure S2(a), the horizontal blue dash line is a little higher when indicating Z. In Supplementary Figure S2(b), Δz (the vertical black dash line) should also be marked in the grey arc region.
In line 174-175, there is one extra closing parenthesis after 0.6s and 1.2s, redspectively.
In line 206, what is the definition of “acquisition time”? In this manuscript, multiple terms make people confused, e.g., exposure time and total time (line 109), total recording time (line 118), exposure setting time (line 122), acquisition time (line 206), actual record time (y-axis in Fig. 1c). It would be helpful to clearly define them, or use figures (e.g., Fig. 1b) to represent them.
In line 233-235, “a fourfold reduction in exposure time (e.g., from 2 s to 0.5 s) using the traditional BIS method does not translate to a fourfold improvement in total data acquisition efficiency.” Neither traditional BIS nor CR-BIS can generate a fourfold improvement in data collection when reducing the exposure time from 2s to 0.5s. As shown in Fig. 1b, CR-BIS-SPA’s EAR has reached 90%, leaving very limited room for further improvement. Is it possible to reach a fourfold improvement in the data throughput when reducing the exposure time from 2s to 0.5s?
In line 250-251, “… data were collected from yeast lamellae at a pixel size of 1.57 Å per tilt series”. “per tilt series” can be removed.
In Fig. 3, Fig. 3d is not labeled in Fig. 3. In its figure legend, the numbers of particles for apoferritin dataset (14k) and ribosome dataset (3.24k) are not correct. They should be 148.7k particles and 32.5k particles, respectively.
In Fig. 4, the typical montage map of lamella shown in (a) is blurry, please update it to a high-quality image. In its figure legend, it states “(b) Defocus variation across tilt angles in one tilt series.” What does x-axis mean in (b)? What is “tomogram lable (or label?)”? Which represent tilt angles? Which represents one tilt series? My understanding is that each number in x-axis indicates one tilt series, so there are 20 tilt series in total. The blue points corresponding to one tilt series indicate the defocus values of different tilt angles. The x-axis of (c) and (d) has the same problem.

Supplementary Figure S5 and Supplementary Figure S6 have the same problem as Fig. 4 in the label of x-axis.
In Supplementary Figure S5, if each number in x-axis indicates one tilt series, there are only 3 tilt series collected by normal BIS. Are 3 tilt series too few to show the tilt alignment? Supplementary Figure S5 is supposed to show the alignment parameter of lamellae-sample collected by normal BIS. However, it shows "X/Y-shift ranges of tilt series acquired with CR-BIS-Tomo" in its figure legend.
In line 300, "Quantitative analysis (Fig. 1b) further reveals that different detectors and frame rates affect the theoretical acceleration factor of CR-BIS". I think it should be Fig. 1c, not Fig. 1b here.
In line 305-306, "At 0.1 s/frame, up to 100 positions (2 s per target) could be collected reliably, whereas at 0.05 s/frame, only 25 positions were stable under similar conditions". What is the underlying reason?
In line 374-375, "For benchmarking speed improvements, control datasets of SPA and tomographic tilt series acquisition were collected using conventional beam-image shift (BIS)". What are the results of the control dataset of SPA and tomography data?
In Supplementary Table 2, what is the lamella milling angle of the ribosome dataset? What is the tilt range of the tilt series? Moreover, both SPA and tomography datasets were collected using Falcon 4i camera. Is there any SPA and tomography results collected using Gatan K3 camera? And what are the results of CR-BIS data collected on Gatan K3?
In line 394, it shows "total dose 120e/A2". However, Supplementary Table 2 shows total dose 123e/A2. Please keep them consistent.
In the Reference, some publications have the DOI link, some don't have. Please keep the format consistent.

(Remarks on code availability)

Reviewer #2

(Remarks to the Author)
In their work, Yang and colleagues address one of the major practical limitations in cryo-EM – data acquisition throughput. Their continuous recording acquisition method more than doubles the acquisition throughput of both single particle analysis and cryo-tomography. This is a tremendous improvement that will translate into proportional increase in productivity of cryo-EM facilities. The paper will be of great interest to the growing community of researchers who utilize cryo-EM in their studies, and I strongly recommend its publication in Nature Communications.

1. Was the use of SerialEM's EarlyReturnNextShot command considered when calculating the K2 and K3 cameras' throughput with standard BIS acquisition?
2. It would be informative to explain the main sources of the intercept term (sensor and driver initialization, etc.) and the slope (frame processing and saving, etc.) terms in Fig. 1c.
3. Simply removing the first frame if it is partially exposed seems a bit wasteful. What are the practical options for perfectly synchronizing the beam blank/unblank with the camera frames?
4. I strongly recommend depositing the CR-BIS SPA and cryo-ET datasets to EMPIAR to facilitate the quick adoption of the method.
5. The manuscript would benefit from language proofreading.

Minor points:
- Abstract – "Data acquisition is always a burden …". A better qualification would be "Cryo-EM data acquisition is time-intensive …".
- The formulas on lines 173 and 174 contain an unnecessary extra closing bracket.
- Fig. 3 caption – a) "14k particles" -> "149k particles", b) "3.2k particles" -> "32.5k particles".
- Fig. S1 caption – "K22 camera" -> "K2 camera".
- Figs 4, S5, and S6 – horizontal axes title "Tomogram Lable" -> "Tomogram Label".
- Line 254 – there is no Fig. 3d panel.
- Cite the PDB accession codes of the atomic models that were fitted in the maps in Fig. 3.

Radostin Danev

(Remarks on code availability)

Reviewer #3

(Remarks to the Author)
The authors proposed a novel approach of continuous recording beam-image shift (CR-BIS) for high-throughput cryo-EM data collection. CR-BIS significantly improves cryo-EM data acquisition efficiency compared to the conventional BIS method. The new method could be of great interest for cryo-EM users. The paper is probably publishable, but should be reviewed again in revised form before it is accepted.

There are three major concerns:

1. "blanking, beam shifting, unblanking" procedure during the exposure could affect data quality to some extent. The authors claim it does not compromise data quality by two test data sets which is not very convincing. The best way to test it is to collect two data sets by using conventional and the CR-BIS methods respectively on the same grids and compare the structure resolutions with the same number of images. If the CR-BIS method yields slightly lower resolution with the same number of images, it is still a good method because it can collect much more images in the same time slot.

2. According to Figure 2a, Ttotal = Tset+Tslope+Tintercept in the exposure sequence. But the actual exposure sequence could be Ttotal = Tintercept1+Tset+Tslope+Tintercept2, where Tintercept1 could be the pre-exposure or exposure preparation time. Which sequence is correct might be refined.

3. Line 303-307
Please explain: (1) why frame rate determines the maximum stable recording time? (2) why only up to 100 or 25 positions could be collected reliably? Is it due to the file size limit and what is the size threshold?

There are some minor issues:

Line 18, "Combine with K3 and Falcon 4i direct detectors"
should be changed to "When combined with K3 and Falcon 4i direct detectors" or "In combination with K3 and Falcon 4i direct detectors".

Line 21, "Further data analysis and reconstructions on both CR-BIS single particle data and tilt series data indicates CR-BIS accelerates data collection without compromising the data quality."
"on both" should be changed to "of both". "indicates" should be changed to "indicate". Actually this sentence can be further refined.

Line 71
"300 tilt series" should be "300 tilt series per day"

Line 131, "We quantified the relationship between Ttotal and Tset, the variation of actual record time with the change of set exposure time of different cameras and frame rate were shown in Fig. 1c."
"Tset, the" should be changed to "Tset. The". "were shown" should be changed to "was shown". Actually this sentence can be further refined.

Line 137
"Higher frame rate is benefit for motion correction" should be changed to "Higher frame rate benefits for motion correction"

Line 136-137
How to define frame rate? If 0.1 s/frame and 0.05 s/frame are frame rates, then it is not higher but lower frame rate (i.e., 0.05 s/frame) which benefits for motion correction but also caused larger slope term according to Fig1. C. Actually 0.1 s and 0.05 s should be frame time and the corresponding frame rates are 10 and 20 frames/s, respectively. With this definition, the declaration "Higher frame rate benefits for motion correction but also caused larger slope term" is correct.

Line 139 "Falcon4i quoted at ~250 fps (frame per second)"
(a) fps (frame per second) should be mentioned at its first appearance. i.e. line 135.
(b) Here is the fact: Falcon4 quoted at ~250 fps while Falcon4i quoted at ~320 fps.

Line 144
Tintercept and Tslope should be clearly mentioned here. For example
The results indicated that Tdelay during data collection includes both slope (Tslope) and intercept terms (Tintercept)
or
The results indicated that Tdelay is the sum of slope term (Tslope) and intercept term (Tintercept).

Line 145-146
This sentence should be well addressed.

Line 150
"exist" should be "existence". This sentence should be rewritten if Tintercept is defined in line 144

Line 154
"take" should be "taking"

Line 154-155 "However, the Ttotal will still increase because of the delay time caused by slope term (Tslope), the horizontal stripes region in Fig. 2a)."
(a) This sentence should be rewritten if Tslope is defined in line 144
(b) "(" is missing corresponding Fig. 2a)

Line 173-175
"(" is missing in two formulae

Line 215
"with the decrease the set exposure time per point" should be changed to "with the decrease of the set exposure time per point"

Line 217
"more detail test parameters" should be changed to "more detailed test parameters"

Line 220
"require" should be "requires"

Line 248
"particles, Fig. 3a" should be changed to "particles (Fig. 3a)" or "particles as shown in Fig. 3a"

Line 254
There is no Fig. 3d in Fig. 3 and no caption for Fig. 3d in Fig. 3.

Line 307
"For the Falcon 4i" should be changed to "For the Falcon 4i detector"

Line 409-410
"re-extract" should be "re-extracted"

Line 420
"3.14Å" should be "3.14 Å"

Figure caption for Fig. S1
"K22" should be "K2"

(Remarks on code availability)

Version 1:

Reviewer comments:

Reviewer #1

(Remarks to the Author)
In the revised manuscript, the authors have resolved the problems and answered the questions I pointed out in my previous review.
There are a few minor comments on the revised manuscript.

In line 177-178 of the revised manuscript, the commonly used cryo-ET acquisition conditions are tilt step of 2° and 3°, tilt range of ±38° and ±60°, and dose of 3 and 4 e−/Å2 per tilt. These tilt ranges are also shown in Supplementary Table 3. The tilt range of ±60° is very typical in the cryo-ET data collection. Does the tilt range of ±38° in this study lead to issues of big missing wedge?

In line 244-245 of the revised manuscript, “under a constant total electron dose, the exposure time required per image scales with the pixel area.” If we want the exposure time scales with the pixel area, it should be under a constant total electron dose (e−/Å2) and a constant dose rate in electrons per physical pixel per second (eps).

In line 274-275 of the revised manuscript, “identical numbers of particles (18,258) were retained, which yielded reconstructions at resolutions of 2.02 Å (BIS-Tomo) and 1.98 Å (CR-BIS-Tomo)”. However, Fig. 3c’s figure legend (line 736-737) shows “both reconstructions were generated from ~23.6k”. And Fig. 3d shows the resolution of BIS-tomo is 2.01 Å (light grey), not 2.02 Å. Please keep the particle number and resolution consistent.

(Remarks on code availability)

Reviewer #2

(Remarks to the Author)
The authors have adequately addressed the reviewers' concerns and suggestions and I recommend the paper for publication.

(Remarks on code availability)

Reviewer #3

(Remarks to the Author)
The major concerns and minor points have been well addressed in the revised manuscript, and I strongly recommend its publication in Nature Communications.

(Remarks on code availability)

**Open Access** This Peer Review File is licensed under a Creative Commons Attribution 4.0 International License, which permits use, sharing, adaptation, distribution and reproduction in any medium or format, as long as you give appropriate credit to the original author(s) and the source, provide a link to the Creative Commons license, and indicate if changes were made.
In cases where reviewers are anonymous, credit should be given to 'Anonymous Referee' and the source.
The images or other third party material in this Peer Review File are included in the article's Creative Commons license, unless indicated otherwise in a credit line to the material. If material is not included in the article's Creative Commons license and your intended use is not permitted by statutory regulation or exceeds the permitted use, you will need to obtain permission directly from the copyright holder.
To view a copy of this license, visit https://creativecommons.org/licenses/by/4.0/

**Response to Reviewers**

We thank the Editor and the Reviewers for their careful evaluation of our manuscript and for their constructive and insightful comments. We have revised the manuscript accordingly, and we believe that the changes have significantly improved the clarity, rigor, and impact of the work. Below, we provide a point-by-point response to all comments. Reviewer comments are reproduced in **bold**, followed by our responses.

**Reviewer #1**

**Due to the high cost and the limited instrument time of cryo-EM, it remains a crucial objective to enhance data collection efficiency in order to push the resolution of the reconstruction. In this study, the authors present CR-BIS framework which eliminates camera-induced delay time by continuously recording during beam-image shift acquisition. CR-BIS enables a significant increase in the SPA and cryo-ET data collection without compromising the data quality, indicating a robust and scalable solution for high-throughput SPA and cryo-ET acquisition.**

1. **In Fig. 1a, the EAR is defined as the ratio of the total recording time (dash blue area) to the total time (all areas). In Fig. 1b, the EAR is defined as the ratio of the exposure setting time (dark blue area) to the total time (all areas), and this study uses the second way to define EAR. If yes, the authors should clearly state this difference in the main text or the figurer legend.**

   We thank the reviewer for pointing out this important ambiguity. We agree that two different efficiency metrics were implicitly used in the original version, which could lead to confusion. In the revised manuscript, we now explicitly distinguish between **conventional acquisition efficiency** and the **effective acquisition ratio (EAR)**. Specifically, the conventional acquisition efficiency refers to the ratio between the total camera recording time (including camera-induced delays) and the total acquisition time, as illustrated in Fig. 1a. In contrast, the EAR is defined as the ratio

between the exposure setting time ($T_{set}$) and the total acquisition time, explicitly excluding camera-related delay, as shown in Fig. 1b.

These definitions have been clarified in the main text, and the descriptions of Fig. 1a and Fig. 1b have been revised accordingly to avoid ambiguity.

2. **In Fig. 1c, please clearly label x-axis and y-axis with Tset and Ttotal.**

   We thank the reviewer for pointing this out. We have revised Fig. 1c and labeled the x-axis and y-axis as $T_{set}$ and $T_{total}$, respectively.

3. **In line 118, "shown as the dash area in Fig1.a". It should be "Fig. 1a".**

   We thank the reviewer for pointing this out. The typo has been corrected, and "Fig1.a" has been revised to "Fig. 1a" in the updated manuscript.

4. **In line 139, Falcon 4i camera internal frame rate is 320 fps, Falcon 4 is 250 fps. The authors cited two references ([12] and [23]) here, but both references used Falcon 4 camera, not Falcon 4i.**

   We thank the reviewer for pointing out this important issue. In the original submission, the camera we used was indeed the Falcon 4 rather than the Falcon 4i. We have incorporated new data acquired using the Falcon 4i direct electron detector, including the corresponding $T_{set}$-$T_{total}$ relationships and apo-ferritin datasets collected using both CR-BIS and BIS on the Falcon 4i direct electron detector. In addition, a reference that explicitly reports the use of the Falcon 4i direct electron detector has been added. In the revised manuscript, we now explicitly state that the Falcon 4 internal frame rate is ~250 fps, while the Falcon 4i internal frame rate is ~320 fps.

5. **In Supplementary Figure S1, it should be K2 camera, not K22 camera.**

   We thank the reviewer for pointing this out. The typo has been corrected, and "K22" has been revised to "K2" in the updated Supplementary Fig. 1.

6. **In line 158-159, "… control stage movement ahead during the slope-derived delay at the end of long-recording …". In Fig. 2a, the intercept term and slope term are connected between each other in CR-BIS method. Does this method only control stage movement ahead during the slope-derived delay, not including the intercept-derived delay?**

   We thank the reviewer for this important question. In practice, stage movement is triggered immediately after completion of a group of target acquisitions, rather than being restricted to a narrowly defined temporal window corresponding solely to the slope-derived delay.

   In Fig. 2a, we divide $T_{delay}$ into an exposure-time-dependent component ($T_{slope}$) and an exposure-time-independent component ($T_{intercept}$). However, we cannot precisely assign individual physical contributors to either component within a single acquisition cycle. As a result, CR-BIS does not selectively operate only during the slope-derived delay while excluding the intercept-derived delay. Instead, advancing stage movement improves utilization of the overall delay period. The largest benefit arises from mitigating the slope-derived component, which increases with longer exposure setting times.

   We agree that the original wording could be misleading and the revised text now states: "*To mitigate the impact of $T_{slope}$, we implemented a script that triggers stage movement ahead at the end of long recordings when the camera and control software remain unresponsive owing to $T_{delay}$. This strategy effectively utilizes the overall delay period, with particular benefit for the exposure-time-dependent $T_{slope}$ component.*"

   "

7. **In line 166, when using the first five tilts to calculate Δz without CR mode, is this universal for any tilt angle, step size, or any dose per tilt?**

   We thank the reviewer for raising this important question. The first five tilts represent the minimum number of non-CR tilts that we recommend for estimating ΔZ and are the parameters used throughout this study. Under commonly used cryo-

ET acquisition conditions (tilt step of 2° and 3°, tilt range of ±38° and ±60°, and dose of 3 and 4 $e^{-}/Å^2$ per tilt), alignment results from the first five tilts consistently provided reliable and stable ΔZ estimation as we tested.

This choice is not intended to be universal for all acquisition schemes. In our implementation, the number of initial non-CR tilts is fully user-configurable in the provided scripts and can be increased if required, for example when using larger tilt range, smaller tilt step. We have clarified this point in the revised manuscript.

8. **In Supplementary Figure S2(a), the horizontal blue dash line is a little higher when indicating Z. In Supplementary Figure S2(b), Δz (the vertical black dash line) should also be marked in the grey arc region.**

   We thank the reviewer for pointing this out. We have modified Supplementary Fig. 2.

9. **In line 174-175, there is one extra closing parenthesis after 0.6s and 1.2s, respectively.**

   We thank the reviewer for pointing this out. The typo has been corrected.

10. **In line 206, what is the definition of "acquisition time"? In this manuscript, multiple terms make people confused, e.g., exposure time and total time (line 109), total recording time (line 118), exposure setting time (line 122), acquisition time (line 206), actual record time (y-axis in Fig. 1c). It would be helpful to clearly define them, or use figures (e.g., Fig. 1b) to represent them.**

    We thank the reviewer for pointing out this ambiguity. We agree that the terminology describing different time components was inconsistent in the original manuscript. In the revised version, we have systematically unified and clarified all time-related definitions, which are now explicitly illustrated in Fig. 1a and Fig. 1b. Specifically, total acquisition time refers to the entire duration required to complete data collection, including camera recording, stage movement and stabilization, and additional operations such as focusing, tracking, and beam-image shift. Total

camera recording time ($T_{total}$) refers exclusively to the camera-related recording period and consists of the exposure setting time ($T_{set}$) and the camera delay time ($T_{delay}$).

The term "actual record time" previously used in Fig. 1c has been replaced with "total camera recording time" to maintain consistency. These revisions have been applied throughout the manuscript and figure labels to avoid confusion.

**11. In line 233-235, "a fourfold reduction in exposure time (e.g., from 2 s to 0.5 s) using the traditional BIS method does not translate to a fourfold improvement in total data acquisition efficiency." Neither traditional BIS nor CR-BIS can generate a fourfold improvement in data collection when reducing the exposure time from 2s to 0.5s. As shown in Fig. 1b, CR-BIS-SPA's EAR has reached 90%, leaving very limited room for further improvement. Is it possible to reach a fourfold improvement in the data throughput when reducing the exposure time from 2s to 0.5s?**

We thank the reviewer for this insightful comment and agree with the reviewer's assessment. Within a given acquisition mode (either conventional BIS or CR-BIS), simply reducing the exposure time from 2 s to 0.5 s cannot yield a fourfold improvement in total data throughput.

The fourfold factor refers to a theoretical requirement when moving to higher magnification, not an expected experimental speedup. Reducing the pixel size from 1.0 Å to 0.5 Å decreases the imaged field of view by a factor of four, requiring four times more images to sample an equivalent area. Under a constant total electron dose, the exposure time per image can be reduced proportionally (e.g., from 2 s to 0.5 s), which would preserve overall throughput only in an ideal, delay-free system. In practice, camera-induced delays limit this scaling. As shown in Supplementary Table 1, for 100 targets acquired using conventional BIS (K3, 0.1 s/frame), reducing the exposure time from 2 s to 0.5 s reduces the total acquisition time by only a factor of 1.46. Under identical conditions, CR-BIS achieves a larger improvement (factor of 2.59), but still falls short of the theoretical value.

The remaining gap arises from (i) per-group focusing overhead, (ii) beam-blanking and beam-image-shift overhead embedded in the exposure setting time of CR mode, and (iii) exposure-time–dependent slope-derived camera delays. We have revised the manuscript to explicitly report these measured ratios rather than implying a fourfold gain, and we have added a discussion of the remaining limitations for CR-BIS-SPA and possible optimization strategies.

12. **In line 250-251, "… data were collected from yeast lamellae at a pixel size of 1.57 Å per tilt series". "per tilt series" can be removed.**

We thank the reviewer for pointing this out. The "per tilt series" has been removed in the update manuscript.

13. **In Fig. 3, Fig. 3d is not labeled in Fig. 3. In its figure legend, the numbers of particles for apoferritin dataset (14k) and ribosome dataset (3.24k) are not correct. They should be 148.7k particles and 32.5k particles, respectively.**

We thank the reviewer for pointing this out. The typo of particle number has been revised in the updated manuscript.

14. **In Fig. 4, the typical montage map of lamella shown in (a) is blurry, please update it to a high-quality image. In its figure legend, it states "(b) Defocus variation across tilt angles in one tilt series." What does x-axis mean in (b)? What is "tomogram lable (or label?)"? Which represent tilt angles? Which represents one tilt series? My understanding is that each number in x-axis indicates one tilt series, so there are 20 tilt series in total. The blue points corresponding to one tilt series indicate the defocus values of different tilt angles. The x-axis of (c) and (d) has the same problem.**

We thank the reviewer for pointing this out. We have replaced the montage map in Fig. 4a with a higher-resolution image. We revised the axis labels and figure legend to explicitly clarify the meaning of the x-axis in Fig. 4b–d. The x-axis now denotes the tilt series index, with each cluster corresponding to one tilt series, while

individual data points represent measurements from different tilt angles within that tilt series. These clarifications have been added to the figure legend to improve readability and avoid ambiguity.

15. **Supplementary Figure S5 and Supplementary Figure S6 have the same problem as Fig. 4 in the label of x-axis.**

    We thank the reviewer for pointing this out. We have revised the figure legends to improve readability and avoid ambiguity.

16. **In Supplementary Figure S5, if each number in x-axis indicates one tilt series, there are only 3 tilt series collected by normal BIS. Are 3 tilt series too few to show the tilt alignment? Supplementary Figure S5 is supposed to show the alignment parameter of lamellae-sample collected by normal BIS. However, it shows "X/Y-shift ranges of tilt series acquired with CR-BIS-Tomo" in its figure legend.**

    We thank the reviewer for the valuable suggestions. In the revised manuscript, we have acquired an additional control dataset using conventional BIS-Tomo on lamellae, replaced the original supplementary figure with one containing a substantially larger number of tilt series, and corrected the figure legend accordingly. The updated alignment analysis is now presented in Supplementary Fig. 6.

17. **In line 300, "Quantitative analysis (Fig. 1b) further reveals that different detectors and frame rates affect the theoretical acceleration factor of CR-BIS". I think it should be Fig. 1c, not Fig. 1b here.**

    We thank the reviewer for pointing this out. This mistake has been corrected.

18. **In line 305-306, "At 0.1 s/frame, up to 100 positions (2 s per target) could be collected reliably, whereas at 0.05 s/frame, only 25 positions were stable under similar conditions". What is the underlying reason?**

We thank the reviewer for raising this important question. For the Gatan K3 direct electron detector, operating at higher frame rates produces a substantially larger number of frames for the same exposure setting time. During long-duration, high-frame-rate acquisitions, we observed that once a certain threshold is exceeded, data collection may terminate with a camera error (e.g., "DM error: Caught an exception from call 3160 to DM function"). Under our test conditions, at 0.1 s/frame, CR-BIS acquisition of 100 positions with a total exposure setting time of 222.75 s produced a file of approximately 30.2 GB containing 2,230 frames. At 0.05 s/frame, only 42 positions (93.99 s total exposure) could be collected reliably, yielding a 17.2 GB file with 1,882 frames.

The exact underlying mechanism remains unclear and likely depends on camera hardware design and internal data handling strategies, which are not accessible to users. As resolving this issue would require modifications at the hardware or firmware level, it is beyond the scope of the present study.

We agree that our original wording was imprecise and have revised the manuscript to report these empirical limits explicitly. The revised text now states: "*Under our tested conditions, the maximum stable exposure setting time for the K3 direct electron detector is approximately 220 s at 0.1 s/frame and 90 s at 0.05 s/frame, while acquisition errors may occur beyond this point.*"

**19. In line 374-375, "For benchmarking speed improvements, control datasets of SPA and tomographic tilt series acquisition were collected using conventional beam-image shift (BIS)". What are the results of the control dataset of SPA and tomography data?**

We thank the reviewer for pointing this out. For benchmarking acquisition speed, we initially compared conventional BIS and CR-BIS using datasets collected on amorphous carbon films without biological samples, and the resulting throughput measurements are summarized in the Table1 and Supplementary Table2.

Following the reviewer's recommendation, we have now added protein sample datasets, including apo-ferritin single-particle and tomographic tilt-series data. These datasets were collected using both conventional BIS and CR-BIS under comparable conditions. The results demonstrate that CR-BIS maintains comparable data quality as normal BIS. The added results are presented in Fig. 3 and Supplementary Fig. 5 and described in the revised manuscript.

20. **In Supplementary Table 2, what is the lamella milling angle of the ribosome dataset? What is the tilt range of the tilt series? Moreover, both SPA and tomography datasets were collected using Falcon 4i camera. Is there any SPA and tomography results collected using Gatan K3 camera? And what are the results of CR-BIS data collected on Gatan K3?**

We thank the reviewer for raising these important points. Following the reviewer's suggestion, we have now included both SPA and cryo-ET datasets collected using the Gatan K3 direct electron detector. Apo-ferritin datasets were acquired using both CR-BIS and conventional BIS on the same grid and within the same mesh, with target positions selected in an interleaved manner to minimize systematic bias arising from local ice thickness or grid heterogeneity. The corresponding results are presented in Supplementary Fig. 5. These K3 datasets show that CR-BIS yields data quality comparable to that obtained with conventional BIS, consistent with our observations using the Falcon 4i direct electron detector. All acquisition parameters for the K3 datasets have been added to Supplementary Tables 2 and 3, and the corresponding processing details are described in the Methods section.

In addition, we have updated the metadata for the in situ ribosome dataset. The lamella milling angle was 10°, and the tilt series was collected over a range of ±60°. These parameters are now explicitly reported in the revised Supplementary Table 3.

21. **In line 394, it shows "total dose 120e/A2". However, Supplementary Table 2 shows total dose 123e/A2. Please keep them consistent.**

We thank the reviewer for pointing this out. We have modified the total dose in line

394 into 123 e/Å $^{2}$.

**22. In the Reference, some publications have the DOI link, some don't have. Please keep the format consistent.**

We thank the reviewer for this comment. The reference format has been checked.

**Reviewer #2:**

**In their work, Yang and colleagues address one of the major practical limitations in cryo-EM – data acquisition throughput. Their continuous recording acquisition method more than doubles the acquisition throughput of both single particle analysis and cryo-tomography. This is a tremendous improvement that will translate into proportional increase in productivity of cryo-EM facilities. The paper will be of great interest to the growing community of researchers who utilize cryo-EM in their studies, and I strongly recommend its publication in Nature Communications.**

1. **Was the use of SerialEM's EarlyReturnNextShot command considered when calculating the K2 and K3 cameras' throughput with standard BIS acquisition?**

   We thank the reviewer for this comment. We tested SerialEM's **EarlyReturnNextShot** command during throughput benchmarking. This option can indeed provide a modest speed improvement. For example, using a Gatan K3 direct electron detector with 100 points per group, 0.1 s/frame, and 2 s per target, the CR-BIS acquisition time was 243 s with EarlyReturnNextShot, compared to 254 s without it.

   However, during downstream processing we observed that using EarlyReturnNextShot in continuous acquisition mode could lead to instability, including improper beam-blanking responses. Considering these reliability issues, we chose not to use EarlyReturnNextShot in our benchmarking experiments or in the publicly released scripts, prioritizing robustness and reproducibility.

2. **It would be informative to explain the main sources of the intercept term (sensor and driver initialization, etc.) and the slope (frame processing and saving, etc.) terms in Fig. 1c.**

   We thank the reviewer for this constructive and insightful suggestion. We agree that the intercept and slope terms in Fig. 1c are very likely associated with distinct physical and software-related processes, such as sensor and driver initialization for the intercept term, and frame processing, buffering, and data writing for the slope term.

   We have considered several plausible sources for the slope-derived delay and performed preliminary tests to evaluate whether storage throughput could be a dominant factor. For example, we attempted to increase the effective write speed by redirecting camera output files to a virtual RAM disk; however, this intervention did not lead to a measurable reduction in the slope term. This suggests that file I/O speed alone is unlikely to be the sole limiting factor.

   At present, we are unable to unambiguously attribute the intercept and slope terms to specific underlying mechanisms. A definitive determination would require more extensive investigation involving camera firmware, drivers, and hardware buffering behavior, which is beyond the scope of the current study. In the absence of direct experimental evidence, we have therefore refrained from making definitive statements in the main text regarding the exact origins of these terms.

   We agree with the reviewer that a more detailed understanding of these delay components would be valuable and believe this represents an important direction for future investigation.

3. **Simply removing the first frame if it is partially exposed seems a bit wasteful. What are the practical options for perfectly synchronizing the beam blank/unblank with the camera frames?**

   We thank the reviewer for this insightful and constructive comment, and we fully agree that precise synchronization between beam blanking/unblanking and camera frame boundaries is an important issue.

For the Falcon 4–series direct electron detectors, we explored a more refined strategy by directly reading the per-frame dose information embedded in the EER files. In this format, fully blank frames contain zero dose, which allows us to identify and exclude black frames with high accuracy while preserving all effective electron exposure.

For K-series cameras, only integrated TIFF frames are available, and frame splitting must rely on intensity information from already accumulated counting results, which inherently limits precision compared with EER-based approaches. Users may configure a relatively low intensity threshold (such as 0.001) to minimize electron dose consumption. More systematic solutions—such as modifying camera drivers or firmware to remove black frames prior to file output—may provide more elegant synchronization. However, implementing such changes would require access to camera hardware and software and is beyond the scope of the present study.

Importantly, comparative tests on real protein samples (updated Fig. 3 and Supplementary Fig. 5) indicate that, for Falcon 4, Falcon 4i, and Gatan K3 direct electron detectors, the proposed frame-handling strategy does not measurably compromise data quality. We therefore believe this approach represents a practical and reliable solution under current hardware constraints.

4. **I strongly recommend depositing the CR-BIS SPA and cryo-ET datasets to EMPIAR to facilitate the quick adoption of the method.**

   We thank the reviewer for this valuable suggestion. We fully agree that public deposition of CR-BIS SPA and cryo-ET datasets will facilitate rapid adoption of the method. We are currently preparing the datasets for EMPIAR submission. Due to the required time for data curation and upload, we chose to first resubmit the revised manuscript and will update the EMPIAR accession information as soon as it becomes available.

5. **manuscript would benefit from language proofreading.**

We thank the reviewer for this suggestion. The manuscript has now undergone thorough language proofreading by a professional editing service to improve grammar, clarity, and overall fluency.

6. **Abstract – "Data acquisition is always a burden …". A better qualification would be "Cryo-EM data acquisition is time-intensive …".**

   We thank the reviewer for this helpful suggestion. We agree that the original wording was imprecise and have revised the sentence accordingly. The revised text now states: "*Cryo-electron microscopy (cryo-EM) data acquisition is time-intensive given that a large amount of data is required to obtain a high-resolution reconstruction.*"

7. **The formulas on lines 173 and 174 contain an unnecessary extra closing bracket.**

   We thank the reviewer for pointing this out. The typo has been corrected.

8. **Fig. 3 caption – a) "14k particles" -> "149k particles", b) "3.2k particles" -> "32.5k particles".**

   We thank the reviewer for pointing this out. The mistake of particle number has been revised in the updated manuscript.

9. **Fig. S1 caption – "K22 camera" -> "K2 camera".**

   We thank the reviewer for pointing this out. The typo has been corrected, and "K22" has been revised to "K2" in the updated Supplementary Figure 1.

**Figs 4, S5, and S6 – horizontal axes title "Tomogram Lable" -> "Tomogram Label".**

We thank the reviewer for pointing this out. We have revised the axis labels and figure legend to explicitly clarify the meaning of the x-axis in Fig. 4b–d. The x-axis

now denotes the tilt series index, with each cluster corresponding to one tilt series, while individual data points represent measurements from different tilt angles within that tilt series. These clarifications have been added to the figure legend to improve readability and avoid ambiguity.

**10. Line 254 – there is no Fig. 3d panel.**

We thank the reviewer for the careful reading of the manuscript and apologize for this oversight. In the revised version, Figure 3 has been updated following the reviewer's suggestions, including the addition of new apo-ferritin single-particle and tomographic quality comparisons. Correspondingly, the figure panels and all references to Figure 3 in the main text have been corrected to ensure consistency.

**11. Cite the PDB accession codes of the atomic models that were fitted in the maps in Fig. 3.**

We thank the reviewer for pointing this out. As requested, the relevant PDB accession codes (PDB-6Z9E[1] for human apo-ferritin and PDB-8UX8[2] for yeast ribosome) have now been cited.

**Reviewer #3:**

**The authors proposed a novel approach of continuous recording beam-image shift (CR-BIS) for high-throughput cryo-EM data collection. CR-BIS significantly improves cryo-EM data acquisition efficiency compared to the conventional BIS method. The new method could be of great interest for cryo-EM users. The paper is probably publishable, but should be reviewed again in revised form before it is accepted.**

1. **"blanking, beam shifting, unblanking" procedure during the exposure could affect data quality to some extent. The authors claim it does not compromise**

**data quality by two test data sets which is not very convincing. The best way to test it is to collect two data sets by using conventional and the CR-BIS methods respectively on the same grids and compare the structure resolutions with the same number of images. If the CR-BIS method yields slightly lower resolution with the same number of images, it is still a good method because it can collect much more images in the same time slot.**

We thank the reviewer for this important and constructive suggestion. We fully agree that a more rigorous, controlled comparison is required to convincingly demonstrate that CR-BIS does not compromise data quality.

Following the reviewer's recommendation, we performed systematic control experiments using apo-ferritin samples collected on Falcon 4i, Falcon 4, and Gatan K3 direct electron detectors. For each camera, datasets were acquired using both conventional BIS and CR-BIS methods. To minimize confounding factors such as ice thickness variations, data for the control and experimental conditions were collected alternately from neighboring targets on the same mesh.

Based on these additional datasets, we revised the main text accordingly and updated Figure 3 to include the Falcon 4i results. Comparative results for Falcon 4 and Gatan K3 are presented in Supplementary Figure 5, respectively. Across all tested cameras, reconstructions from CR-BIS data show comparable resolution and map quality to those obtained using conventional BIS, providing stronger evidence that CR-BIS does not adversely affect data quality.

We did not perform equivalent control experiments on FIB-milled cellular lamellae, as achieving uniform lamella thickness and comparable target distributions across different lamellae is inherently challenging. Considering these practical limitations, we selected apo-ferritin as a well-controlled benchmark specimen for systematic evaluation.

2. **According to Figure 2a, Ttotal = Tset+Tslope+Tintercept in the exposure sequence. But the actual exposure sequence could be Ttotal =**

**Tintercept1+Tset+Tslope+Tintercept2, where Tintercept1 could be the pre-exposure or exposure preparation time. Which sequence is correct might be refined.**

We thank the reviewer for this insightful comment and agree that this point required clarification. In Fig. 2a, $T_{delay}$ is depicted after $T_{set}$ to more clearly illustrate that CR-BIS reduces the repeated, per-exposure delay component ($T_{intercept}$) that accumulates across recordings. However, as the reviewer correctly points out, in practice the intercept-related delays are likely distributed both before and after the nominal exposure period (e.g., preparation and post-exposure handling).

In the revised manuscript, we have explicitly clarified that the schematic in Fig. 2a is not intended to represent the exact temporal order of events. Instead, it provides a conceptual decomposition of the total delay into exposure-dependent ($T_{slope}$) and exposure-independent ($T_{intercept}$) components, which may be distributed at different stages of the exposure sequence.

3. **Line 303-307**

**Please explain: (1) why frame rate determines the maximum stable recording time? (2) why only up to 100 or 25 positions could be collected reliably? Is it due to the file size limit and what is the size threshold?**

We thank the reviewer for raising this important question. For the Gatan K3 direct electron detector, operating at higher frame rates produces a substantially larger number of frames for the same exposure setting time. During long-duration, high-frame-rate acquisitions, we observed that once a certain threshold is exceeded, data collection may terminate with a camera error (e.g., “DM error: Caught an exception from call 3160 to DM function”). Under our test conditions, at 0.1 s/frame, CR-BIS acquisition of 100 positions with a total exposure setting time of 222.75 s produced a file of approximately 30.2 GB containing 2,230 frames. At 0.05 s/frame, only 42 positions (93.99 s total exposure) could be collected reliably, yielding a 17.2 GB file with 1,882 frames.

The exact underlying mechanism remains unclear and likely depends on camera hardware design and internal data handling strategies, which are not accessible to users. As resolving this issue would require modifications at the hardware or firmware level, it is beyond the scope of the present study.

We agree that our original wording was imprecise and have revised the manuscript to report these empirical limits explicitly. The revised text now states: "*Under our tested conditions, the maximum stable exposure setting time for the K3 direct electron detector is approximately 220 s at 0.1 s/frame and 90 s at 0.05 s/frame, while acquisition errors may occur beyond this point.*"

4. **Line 18, "Combine with K3 and Falcon 4i direct detectors" should be changed to "When combined with K3 and Falcon 4i direct detectors" or "In combination with K3 and Falcon 4i direct detectors".**

   We agree with the reviewer's suggestion for improved phrasing. The revised text now states: "*The utilization of CR-BIS with K3 and Falcon 4 direct electron detectors and conventional data acquisition conditions enables the acquisition of ~34,000 micrographs and ~1,000 tilt series per 24 h in single-particle analysis mode and cryo-electron tomography mode, respectively.*"

5. **Line 21, "Further data analysis and reconstructions on both CR-BIS single particle data and tilt series data indicates CR-BIS accelerates data collection without compromising the data quality.", "on both" should be changed to "of both". "indicates" should be changed to "indicate". Actually this sentence can be further refined.**

   We thank the reviewer for this insightful comment. We have revised this sentence into "*Three-dimensional reconstructions of single-particle and tomographic datasets show that CR-BIS accelerates data collection and maintains data quality.*"

6. **Line 71:"300 tilt series" should be "300 tilt series per day"**

   We thank the reviewer for pointing this out. The mistake has been corrected.

7. **Line 131, "We quantified the relationship between Ttotal and Tset, the variation of actual record time with the change of set exposure time of different cameras and frame rate were shown in Fig. 1c."**

   **"Tset, the" should be changed to "Tset. The". "were shown" should be changed to "was shown". Actually this sentence can be further refined.**

   We thank the reviewer for this constructive comment. We have rewritten the sentence for greater clarity. The revised text now states: "*We quantified the relationship between $T_{set}$ and $T_{total}$. The variation of $T_{total}$ with the change in $T_{set}$ of different cameras and frame times is shown in Fig. 1c.*"

8. **Line 137**

   **"Higher frame rate is benefit for motion correction" should be changed to "Higher frame rate benefits for motion correction"**

   We thank the reviewer for pointing out the grammatical error. The sentence has been corrected to: "*A higher frame rate benefits motion correction …*".

9. **Line 136-137**

   **How to define frame rate? If 0.1 s/frame and 0.05 s/frame are frame rates, then it is not higher but lower frame rate (i.e., 0.05 s/frame) which benefits for motion correction but also caused larger slope term according to Fig1. C. Actually 0.1 s and 0.05 s should be frame time and the corresponding frame rates are 10 and 20 frames/s, respectively. With this definition, the declaration "Higher frame rate benefits for motion correction but also caused larger slope term" is correct.**

   We thank the reviewer for pointing out this important ambiguity. In the revised manuscript, we now explicitly define frame time and frame rate. Specifically, 0.1

s/frame and 0.05 s/frame refer to frame time, corresponding to frame rates of 10 frames/s and 20 frames/s, respectively. With this clarification, the statement "*A higher frame rate benefits motion correction, although it results in a larger slope term (Fig. 1c).*"

**10. Line 139 "Falcon4i quoted at ~250 fps (frame per second)"**

**(a) fps (frame per second) should be mentioned at its first appearance. i.e. line 135.**

**(b) Here is the fact: Falcon4 quoted at ~250 fps while Falcon4i quoted at ~320 fps.**

We thank the reviewer for these precise corrections. We have (a) defined "fps (frames per second)" upon its first appearance in the text, and (b) In the original submission, the camera we used was indeed the Falcon 4 rather than the Falcon 4i. we have incorporated new data acquired using the Falcon 4i direct electron detector, including the corresponding $T_{set}$-$T_{total}$ relationships and apo-ferritin datasets collected using both CR-BIS and BIS on the Falcon 4i direct electron detector. In addition, a reference that explicitly reports the use of the Falcon 4i direct electron detector has been added. In the revised manuscript, we have clarified this distinction and updated the references accordingly. Specifically, we now explicitly state that the Falcon 4 internal frame rate is ~250 fps, while the Falcon 4i internal frame rate is ~320 fps.

**11. Line 144**

**Tintercept and Tslope should be clearly mentioned here. For example: "The results indicated that Tdelay during data collection includes both slope (Tslope) and intercept terms (Tintercept)"**

**or "The results indicated that Tdelay is the sum of slope term (Tslope) and intercept term (Tintercept)."**

We appreciate the reviewer's suggestion for increased clarity. We have revised the

text to explicitly state: “These results indicate that $T_{delay}$ is the sum of the slope term ($T_{slope}$) and intercept term ($T_{intercept}$).” A detailed explanation of these two components is provided in the subsequent section, “The principle and workflow of CR-BIS.”

**12. Line 145-146**

**This sentence should be well addressed.**

We thank the reviewer for highlighting the unclear phrasing. The sentence has been revised for better clarity and now reads: “*Therefore, it is critical to address the camera delay time to enhance the efficiency of cryo-EM data acquisition and improve the EAR.*”

**13. Line 150**

**"exist" should be "existence". This sentence should be rewritten if Tintercept is defined in line 144**

We thank the reviewer for catching this error. In response to the collective comments from the reviewers regarding the explanation of $T_{slope}$ and $T_{intercept}$, we have thoroughly rewritten and reorganized this entire paragraph. The new version provides a more detailed and logically coherent explanation of the slope term ($T_{slope}$) and intercept term ($T_{intercept}$).

**14. Line 154**

**"take" should be "taking"**

We thank the reviewer for pointing this out. The typo has been corrected.

**15. Line 154-155 "However, the Ttotal will still increase because of the delay time caused by slope term (Tslope), the horizontal stripes region in Fig. 2a)."**

**(a) This sentence should be rewritten if Tslope is defined in line 144**

**(b) "(" is missing corresponding Fig. 2a)**

We thank the reviewer for pointing this out. In response to the collective comments regarding the explanation of $T_{slop}$ and $T_{intercept}$, we have comprehensively rewritten and reorganized the entire paragraph for improved logical flow and clarity. The revised text now includes a proper definition of $T_{slop}$ and correct referencing to Fig. 2a.

**16. Line 173-175**

**"(" is missing in two formulae**

We thank the reviewer for pointing this out. The typo has been corrected.

**17. Line 215**

**"with the decrease the set exposure time per point" should be changed to "with the decrease of the set exposure time per point"**

We thank the reviewer for the correction. The phrase has been amended to "*The improvement ratio for K3 and Falcon 4 direct electron detectors increased with the decrease of exposure setting times per point (Supplementary Table 1).*"

**18. Line 217**

**"more detail test parameters" should be changed to "more detailed test parameters"**

We thank the reviewer for catching this error. The phrase has been corrected to "more detailed test parameters".

**19. Line 220**

**"require" should be "requires"**

We thank the reviewer for pointing this out. The phrase has been corrected.

**20. Line 248**

**"particles, Fig. 3a" should be changed to "particles (Fig. 3a)" or "particles as**

**shown in Fig. 3a"**

We thank the reviewer for the correction. Fig. 3a has been updated and this phrase has been revised.

**21. Line 254**

**There is no Fig. 3d in Fig. 3 and no caption for Fig. 3d in Fig. 3.**

We thank the reviewer for identifying this inconsistency. We have updated Fig. 3 and its caption in the revised manuscript to ensure all panel references (a-f) are correctly presented and described.

**22. Line 307**

**"For the Falcon 4i" should be changed to "For the Falcon 4i detector"**

We thank the reviewer for this suggestion. Accordingly, we have not only made the specified change but have also systematically reviewed the entire manuscript to standardize the terminology. All references to these devices (e.g., Falcon 4i, Falcon 4, K3) have been updated to the consistent and precise formulation "direct electron detector" (e.g., "Falcon 4i direct electron detector").

**23. Line 409-410**

**"re-extract" should be "re-extracted"**

We thank the reviewer for pointing this out. The mistake has been corrected.

**24. Line 420**

**"3.14Å" should be "3.14 Å"**

We thank the reviewer for pointing this out. The typo has been corrected.

**25. Figure caption for Fig. S1**

**"K22" should be "K2"**

We thank the reviewer for pointing this out. The typo has been corrected, and “K22”

has been revised to “K2” in the updated Supplementary Figure 1.

## Reference


1. Yip KM, Fischer N, Paknia E, Chari A, Stark H. Atomic-resolution protein structure determination by cryo-EM. *Nature* **587**, 157-161 (2020).

2. Cheng J, Wu C, Li J, Yang Q, Zhao M, Zhang X. Capturing eukaryotic ribosome dynamics in situ at high resolution. *Nature Structural & Molecular Biology* **32**, 698-708 (2025).

**Response to Reviewers**

We thank the Editor and the Reviewers for their careful evaluation of our manuscript and for their constructive and insightful comments. Below, we provide a point-by-point response to all comments. Reviewer comments are reproduced in **bold**, followed by our responses.

**Reviewer #1**

**In the revised manuscript, the authors have resolved the problems and answered the questions I pointed out in my previous review. There are a few minor comments on the revised manuscript.**

1. **In line 177-178 of the revised manuscript, the commonly used cryo-ET acquisition conditions are tilt step of 2° and 3°, tilt range of ±38° and ±60°, and dose of 3 and 4 e−/Å2 per tilt. These tilt ranges are also shown in Supplementary Table 3. The tilt range of ±60° is very typical in the cryo-ET data collection. Does the tilt range of ±38° in this study lead to issues of big missing wedge?**

   We thank the reviewer for this important question. The ±38° tilt range was intentionally chosen for this apoferritin benchmark and does introduce a bigger missing-wedge effect in the tomogram for this dataset comparing with the ±60° dataset. However, a tilt range around ±40° has been used in previous work for sub-tomogram averaging[1,2]. This averaging procedure can minimize the missing-wedge effect on the high-resolution reconstruction.

2. **In line 244-245 of the revised manuscript, "under a constant total electron dose, the exposure time required per image scales with the pixel area." If we want the exposure time scales with the pixel area, it should be under a constant total electron dose (e−/Å2) and a constant dose rate in electrons per physical pixel per second (eps).**

   We thank the reviewer for this important clarification. As the reviewer correctly points out, the exposure time scales with pixel area only under both a constant total electron dose ($e^-/Å^2$) and a constant dose rate in electrons per physical pixel per second (eps). We have revised the sentence accordingly in the manuscript to explicitly include this condition.

**3. In line 274-275 of the revised manuscript, "identical numbers of particles (18,258) were retained, which yielded reconstructions at resolutions of 2.02 Å (BIS-Tomo) and 1.98 Å (CR-BIS-Tomo)". However, Fig. 3c' s figure legend (line 736-737) shows "both reconstructions were generated from ~23.6k". And Fig. 3d shows the resolution of BIS-tomo is 2.01 Å (light grey), not 2.02 Å. Please keep the particle number and resolution consistent.**

We thank the reviewer for carefully identifying these inconsistencies. We have now corrected these values and made them consistent in the main text, Fig. 3 legend, and Fig. 3d. We apologize for the oversight.

## Reference


1. Bouvette J*, et al.* Beam image-shift accelerated data acquisition for near-atomic resolution single-particle cryo-electron tomography. *Nature Communications* **12**, 1957 (2021).

2. Tegunov D, Xue L, Dienemann C, Cramer P, Mahamid J. Multi-particle cryo-EM refinement with M visualizes ribosome-antibiotic complex at 3.5 Å in cells. *Nature Methods* **18**, 186-193 (2021).
